# An AI-powered self-driving microscope for low-cost acute leukemia detection

**DOI:** 10.1038/s41467-026-77130-x

**Published:** 2026-08-27

**Authors:** Ethan S. Yan, Shenghuan Sun, Zhanghan Yin, Ali Kamali, Jacob Van Cleave, Irem S. Isgor, Brenda Fried, Cesar Colorado-Jimenez, Deepika Dilip, Jeeyeon Baik, Allyne Manzo, Sean Paulsen, Siddharth Singi, Scott Drapeau, Ozgur Can Eren, Joanne K. Chun, Aijazuddin Syed, Anthony Cardillo, Melissa Pulitzer, Maly Fenelus, David Kim, Jamal Benhamida, Dianna Ng, Orly Ardon, Mikhael Roshal, Ahmet Dogan, Chad Vanderbilt, Khawaja H. Bilal, Gregory M. Goldgof

**Affiliations:** 1 Department of Pathology and Laboratory Medicine, Memorial Sloan Kettering Cancer Center, New York, NY USA; 2 Bakar Computational Health Sciences Institute, University of California, San Francisco, San Francisco, CA USA; 3 Department of Statistics, University of California, Berkeley, Berkeley, CA USA; 4 The Warren Alpert Center for Digital and Computational Pathology, Memorial Sloan Kettering Cancer Center, New York, NY USA; 5 School of Medicine, New York Medical College, Valhalla, NY USA; 6 Makers Lab, University of California, San Francisco, San Francisco, CA USA; 7 Department of Bioengineering and Therapeutic Sciences, University of California, San Francisco, San Francisco, CA USA; 8 Department of Pathology and Laboratory Medicine, Weill Cornell Medical College, New York, NY USA; 9 Halvorsen Center for Computational Oncology, Memorial Sloan Kettering Cancer Center, New York, NY USA

**Keywords:** Cancer imaging, Pathology, Acute myeloid leukaemia, Cancer screening, Machine learning

## Abstract

Current artificial intelligence systems for leukemia detection typically rely on costly whole-slide scanners, limiting accessibility in low-resource settings. We present ALLocate, a low-cost, artificial intelligence-powered microscope plugin that enables self-driving microscopy for leukemia detection. ALLocate attaches directly to conventional microscopes and provides automated analysis at a fraction of the cost of a whole-slide scanner. We evaluate its robustness at three levels: region-of-interest identification, cell detection, and end-to-end slide-level diagnosis. The system is trained and evaluated using more than 11,000 annotated regions and 130,000 annotated cells and is further validated using independent multi-institutional cohorts, including 165 physical bone marrow smear slides. ALLocate achieves an area under the receiver operating characteristic curve greater than 0.99 for region-of-interest identification, a mean average precision at 50% intersection over union of 0.90 for cell detection, and 88% accuracy for slide-level diagnosis on glass slides without requiring a whole-slide scanner. These results suggest that ALLocate provides an accurate, generalizable, and cost-effective approach for automated bone marrow smear screening, helping bridge the gap between AI innovation and practical deployment in resource-limited settings where access to specialist expertise may be limited.

## Abstract (web summary)

Current AI systems for leukemia detection typically rely on costly whole-slide scanners, limiting accessibility in low-resource settings. Here, the authors present ALLocate, a low-cost, AI-driven microscope plugin that enables self-driving microscopy for accurate and affordable leukemia detection without whole-slide scanners.

## Introduction

Acute myeloid leukemia (AML) is a hematologic malignancy caused by the proliferation of hematopoietic stem cells and accounts for approximately 28% of leukemia-related deaths worldwide^1–3^. Diagnosis typically requires identification of ≥20% leukemic blasts in bone marrow or peripheral blood^4–7^, a process that depends on manual examination of 200–500 cells per smear under light microscopy. This task, typically performed by trained pathologists or hematologists, is time-consuming, labor-intensive, and inherently variable due to its subjective nature^7,8^. As clinical volumes rise, the need for faster, standardized diagnostics grows, especially in resource-constrained settings with limited access to expert hematologists or pathologists. For instance, countries in sub-Saharan Africa have only 0.1 to 1.3 hematopathologists per million people, creating substantial barriers to the timely diagnosis of hematologic malignancies^9^.

Recent deep learning approaches have shown considerable promise in automating bone marrow histologic evaluation, particularly for single-cell classification^10,11^. Matek et al. applied convolutional neural networks (CNNs) to over 170,000 single-cell images from marrow smears, achieving high performance across cell types^12^. Sun et al. introduced a deep ensemble model for bone marrow morphometry that demonstrated expert-level accuracy and strong generalizability on an independent dataset^13^. Incorporating object detection techniques, Chandradevan et al. developed a two-stage pipeline combining Faster R-CNN and VGG16, reporting an AUC-ROC of 96% for detection and 98% for classification using scanned images from 17 patients^14^. Lewis et al. proposed a three-part framework consisting of a region classifier, Fast R-CNN, and CNN-based classification, achieving accuracies ranging from 62% to 96% across various cell types^15^. Nakamura et al. applied semi-supervised learning and segmentation strategies, reaching 98% accuracy in region classification^16^. Beyond region proposal-based models, YOLO architectures have also been explored in hematology; however, most applications have focused on peripheral blood^17,18^. To date, only a limited number of studies have applied YOLO models specifically to bone marrow smears^19,20^.

While these studies have shown encouraging results, most rely on expensive high-end whole-slide imaging (WSI) systems, which are prohibitively expensive, often exceeding $300,000 USD, and are not readily available in many hospitals or small clinics^14,15,20^. This challenge is even greater in low- and middle-income countries (LMICs), where diagnostic infrastructure and specialist availability are severely limited. WSIs enable large-scale data generation and model training but limit deployment due to cost and technical requirements. Manual images captured with light microscopes are more accessible and more common in LMICs’ clinical laboratories, but remain inefficient and impractical for AI deployment due to laborious field selection. This creates a diagnostic gap, where timely leukemia diagnosis is hindered by the lack of affordable imaging tools and automated analysis systems. Overall, an affordable, automated method for capturing and analyzing marrow smears without relying on whole-slide scanners is urgently needed. Despite ongoing advances in deep learning applications to assist in the diagnostic evaluation of leukemia, there remains a need for an AI system that is low-cost, fully automated, and capable of generalizing across real-world smear variability. Recent efforts have explored low-cost and edge-based microscopy platforms for hematologic and cytologic applications. For example, Li et al. developed an autonomous, low-cost microscopy system (~$250–$500) capable of detecting infectious agents in peripheral blood smears, including *Loa loa*, *Trypanosoma brucei rhodesiense*, and malaria^21,22^. Similarly, Maturana et al. introduced a 3D-printed robotic add-on with smartphone-based imaging for malaria detection^23^. While these approaches, together with other low-cost digital microscopy frameworks, demonstrate the feasibility of inexpensive automated microscopy^24,25^, they are primarily focused on infectious disease detection and peripheral blood analysis, with limited evaluation for leukocyte classification in bone marrow smears.

In this work, we present ALLocate (Acute Leukemia Locate), a relatively low-cost, AI-powered self-driving microscope system enabling blast detection for leukemia evaluation. It integrates an inexpensive, 3D-printed programmable microscope scanner with two deep learning modules: a CNN-based region classifier and a YOLOv8-based, one-step cell detection and classification model. Designed to operate without reliance on expensive whole-slide imaging infrastructure, ALLocate enables rapid, accurate, and affordable leukemia diagnosis. We evaluated its performance across three core tasks: ROI identification, blast cell detection, and full slide-level diagnosis and quantification on both digital and glass slides. This study leverages a large-scale annotated dataset comprising more than 11,000 marrow regions and 130,000 individual cells, and includes extensive validation on independent cohorts, including a multi-institutional set of 165 physical bone marrow smear slides. Overall, we evaluated a computational pathology framework across both digital slide analysis and real-time glass slide assessment using a self-driving microscope platform. Our evaluation validates ALLocate's robustness, precision, and generalizability, underscoring its potential as a less costly solution for AI-assisted leukemia diagnosis in resource-limited clinical settings.

## Results

### Cost-friendly AI-driven pipeline for slide evaluation

**Fig. 1:**
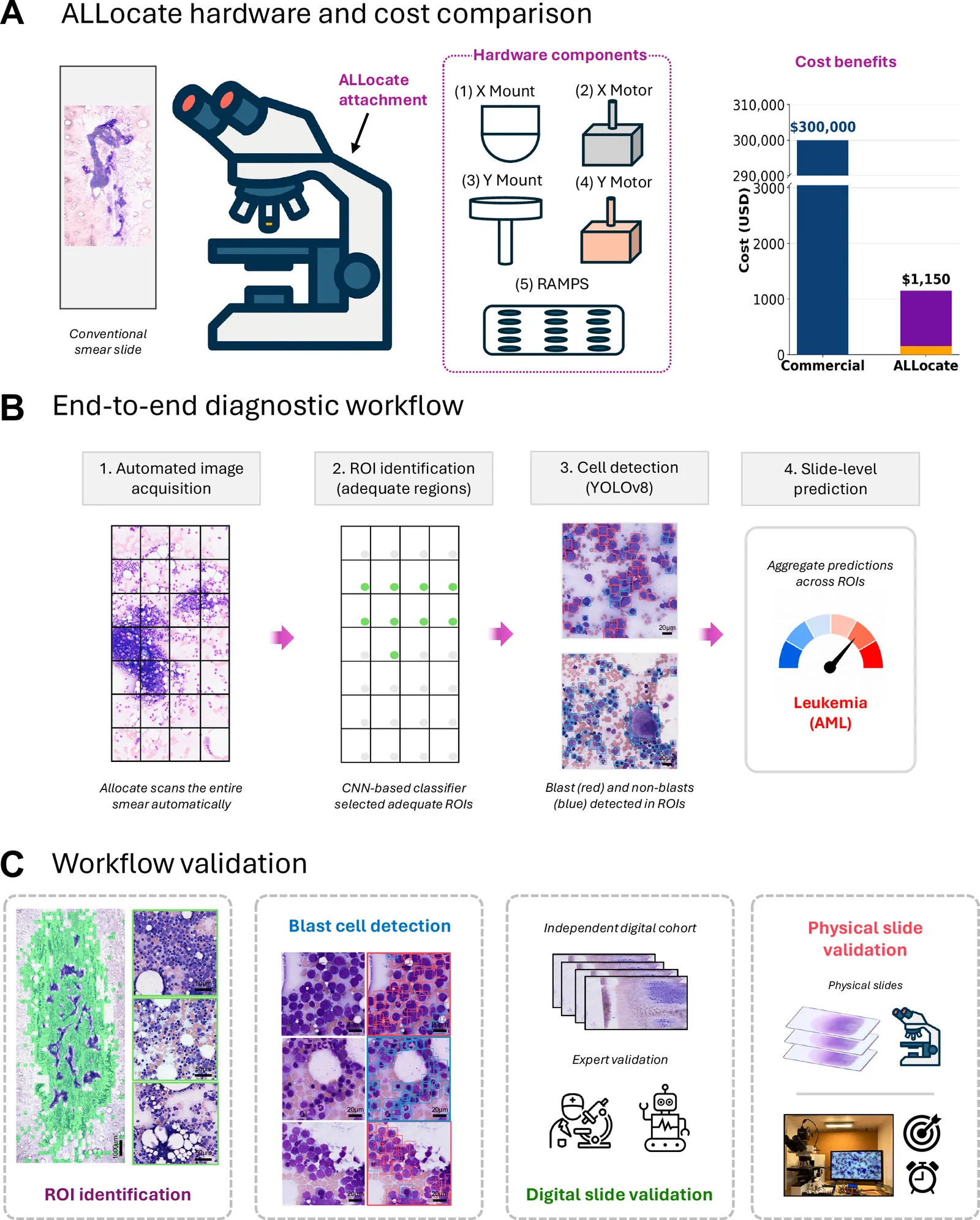
Fully integrated system design of ALLocate. **A** The ALLocate hardware consists of a RepRap Arduino Mega Polulu Shield (RAMPS) control board shield on an Arduino Mega 2560 board (5), 3D-printed parts (1, 3), and two NEMA 14 motors (2, 4). The ALLocate cost ($150, $1150 with microscope) is significantly lower than WSI scanners. **B** The smear is scanned by ALLocate hardware connected to the microscope and then processed through an image sampling system, which segments the image into smaller tiles and passes them through a region classifier. YOLOv8 is applied to the adequate regions, giving precise detection within selected regions. **C** Workflow validation across multiple levels, including region-of-interest identification, blast cell detection, validation on an independent digital-slide cohort, and end-to-end evaluation on physical glass slides.

An integrated deep learning model system was developed for automatic bone marrow slide evaluation without the need to digitize slides using a whole-slide scanner or viewer (Fig. 1). The smear will first pass through the ALLocate Automated Microscope Scanning System (AAMSS), which was implemented in accordance with an existing proof-of-concept design^26^. AAMSS transforms the smears into digital images automatically using stepper motors and a program to automatically control the movement of the microscope stage (Fig. 1A). It consists of two NEMA 14 motors that are mounted into a 3D-printed shell adjacent to the existing knobs for manual control of the microscope stage. Customizable 3D-printed fittings are mounted to the X and Y knobs of the stage and connected to the stepper motors (Supplementary Dataset 1). Through a program, the scanning duration and path can be controlled and uploaded to the Arduino control board, enabling automated capture of a representative region of the smear. Further details on the implementation of AAMSS and its controller are included in the Methods section.

Downstream of the hardware module, the integrated AI modules deliver the diagnostic power of ALLocate. Each of the captured images is passed through a trained region classifier to select the adequate marrow regions. Furthermore, a trained cell detection module is applied on the diagnostic regions, giving a precise detection of blasts and normal cells (Fig. 1B). Model development was performed exclusively using data from the University of California, San Francisco (UCSF), while validation experiments were conducted on independent cohorts from both UCSF and Memorial Sloan Kettering Cancer Center (MSKCC) to ensure robust assessment of generalizability (Table 1 and Table S1).

**Table 1:** Summary of Tasks and Associated Datasets Used in This Study

Dataset Purpose		Inst.	Hardware	Data Type	Tasks	Qty	Patients
TR	Training & Evaluation	UCSF	40x/400x-equivalent WSI images (Aperio AT2, GT450)	Region Tiles	Model development	9,149	70
ER		UCSF	40x/400x-equivalent WSI images (Aperio AT2, GT450)		Adequacy evaluation	2,286	
TC	Training & Evaluation	UCSF	40x/400x-equivalent WSI images (Aperio AT2, GT450)		Model development	109,825	170
EC		UCSF	40x/400x-equivalent WSI images (Aperio AT2, GT450)		Cell classification	26,533	
EVC	External Validation	MSKCC	40x/400x-equivalent WSI images (Hamamatsu S360, S20)		Malignancy evaluation	186	95
EVS		MSKCC	40x/400x-equivalent WSI images (Hamamatsu S360, S20)	Digital Slides	Malignancy evaluation	40	40
SDM	Self Driving Microscope	UCSF	40x/400x self-driving compound microscope (Olympus BX40, 3D printed actuators, Arduino controller)	Glass Slides	Malignancy evaluation	36	36
		MSKCC	40x/400x self-driving compound microscope (Olympus BX46, 3D printed actuators, Arduino controller)		Malignancy evaluation	129	129

*TR* Training set for Region classification, *TC* Training set for Cell detection, *ER* internal Evaluation set for Region classification, *EC* internal Evaluation set for Cell detection, *EVC* External Validation set for Cell-level AI framework, *EVS* External Validation set for Slide-level AI framework, *SDM* evaluation using the Self-Driving Microscope on physical glass slides.

**Fig. 2:**
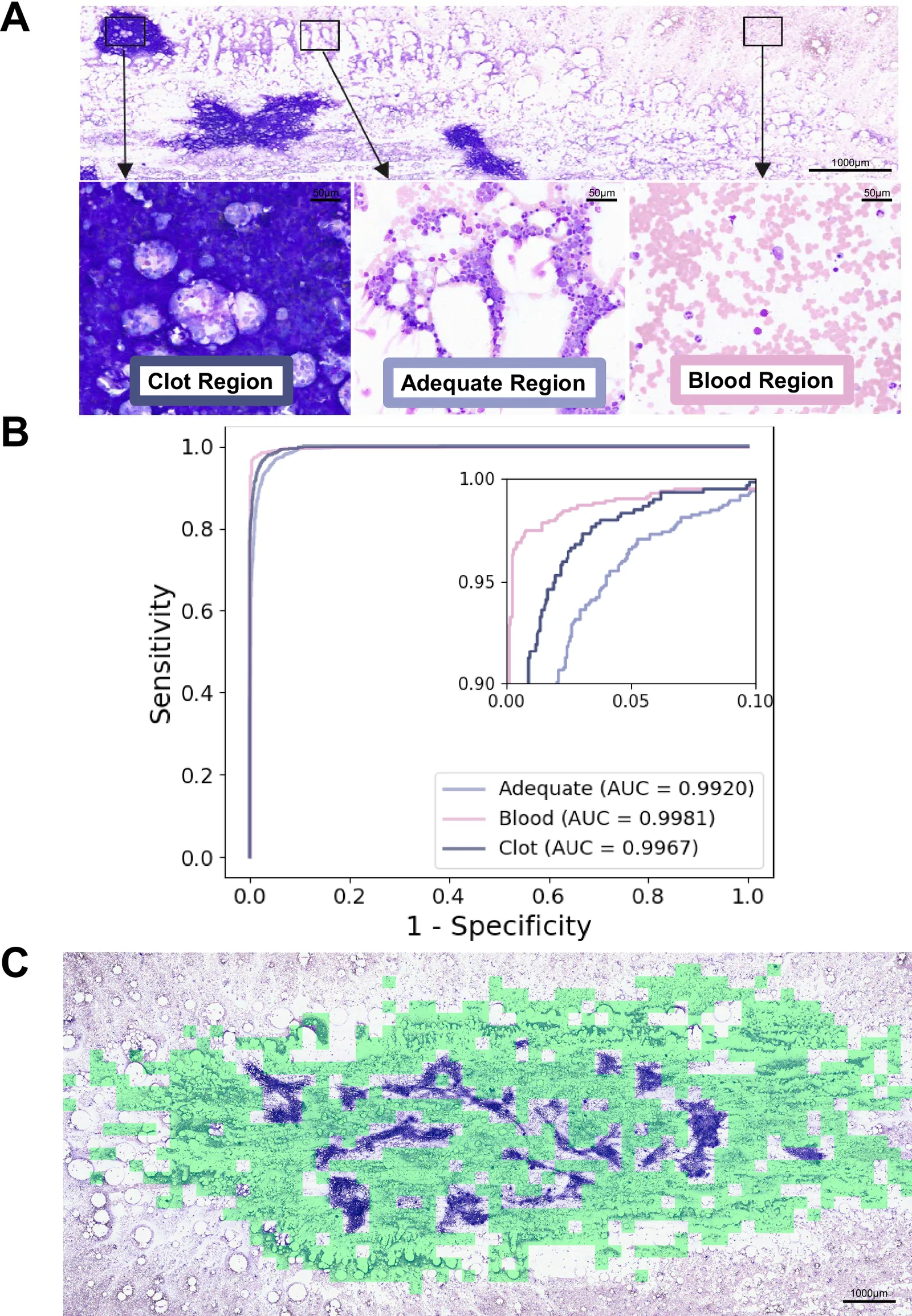
Region classifier development in the marrow aspirate smears. **A** The top image shows a whole-slide image of marrow aspirate smears at 2× magnification. The bottom images show 40× magnification of different regions: clotted regions, adequate regions, and blood regions. **B** ROC curve of the CNN region classifier. **C** Visualization of the region classifier model on the whole smear. Areas with green overlay represent adequate classification.

**Fig. 3:**
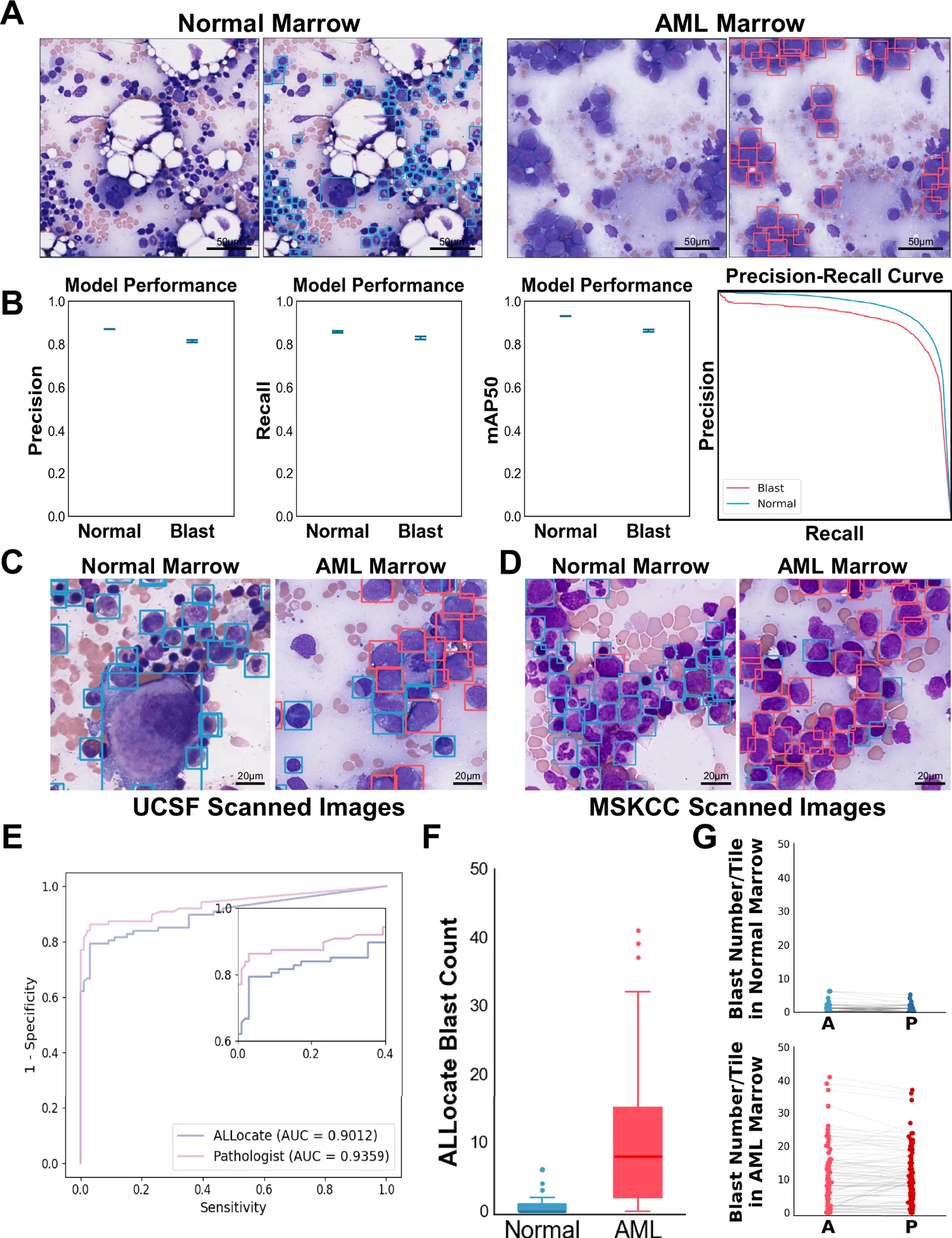
Comprehensive assessment of blast cell detection with in-domain and external evaluation. **A** Marrow cells in an adequate region in normal and AML marrow annotated by bounding boxes (Blue: normal cells; Red: blasts). **B** YOLOv8 performance for detecting normal cells and blasts, including precision, recall, mAP50, and precision-recall curves. Bars represent mean values across five cross-validation folds, and error bars represent ±1 standard deviation, with each fold treated as one independent computational replicate (*n* = 5 folds); corresponding 95% confidence intervals are: precision [0.864–0.870], recall [0.849–0.867], and mAP50 [0.926–0.928] for normal cells, and precision [0.805–0.821], recall [0.816–0.844], and mAP50 [0.856–0.870] for blasts. **C** Real-time detection of normal and leukemia marrow regions from the UCSF database (Blue: normal cells; Red: blasts). **D** Real-time detection of normal and leukemia marrow regions from the MSKCC database (Blue: normal cells; Red: blasts). **E** ROC curves comparing ALLocate and pathologist performance for region-level AML classification on the external cell-level validation cohort (EVC). Ground-truth labels were curated by expert hematopathologists. Prediction scores were defined as blast ratios (blast cells / total nucleated cells) for both the model and an independent pathologist. **F** Blast count distribution of ALLocate on the normal and AML tiles from MSKCC (EVC). Box plots show the median as the center line, the 25th and 75th percentiles as the lower and upper bounds of the box, and whiskers extending to the most extreme values within 1.5 times the interquartile range (mean: 0.7 normal, 10.5 AML). Individual points represent individual regions. The analysis included 99 normal regions and 87 AML regions (*n* = 186 regions), with each region treated as the unit of analysis. **G** Paired swarm plot of blast counts produced by ALLocate (A) on normal and AML regions paired with the count of the pathologist (P). Source data are provided as a Source Data file.

**Fig. 4:**
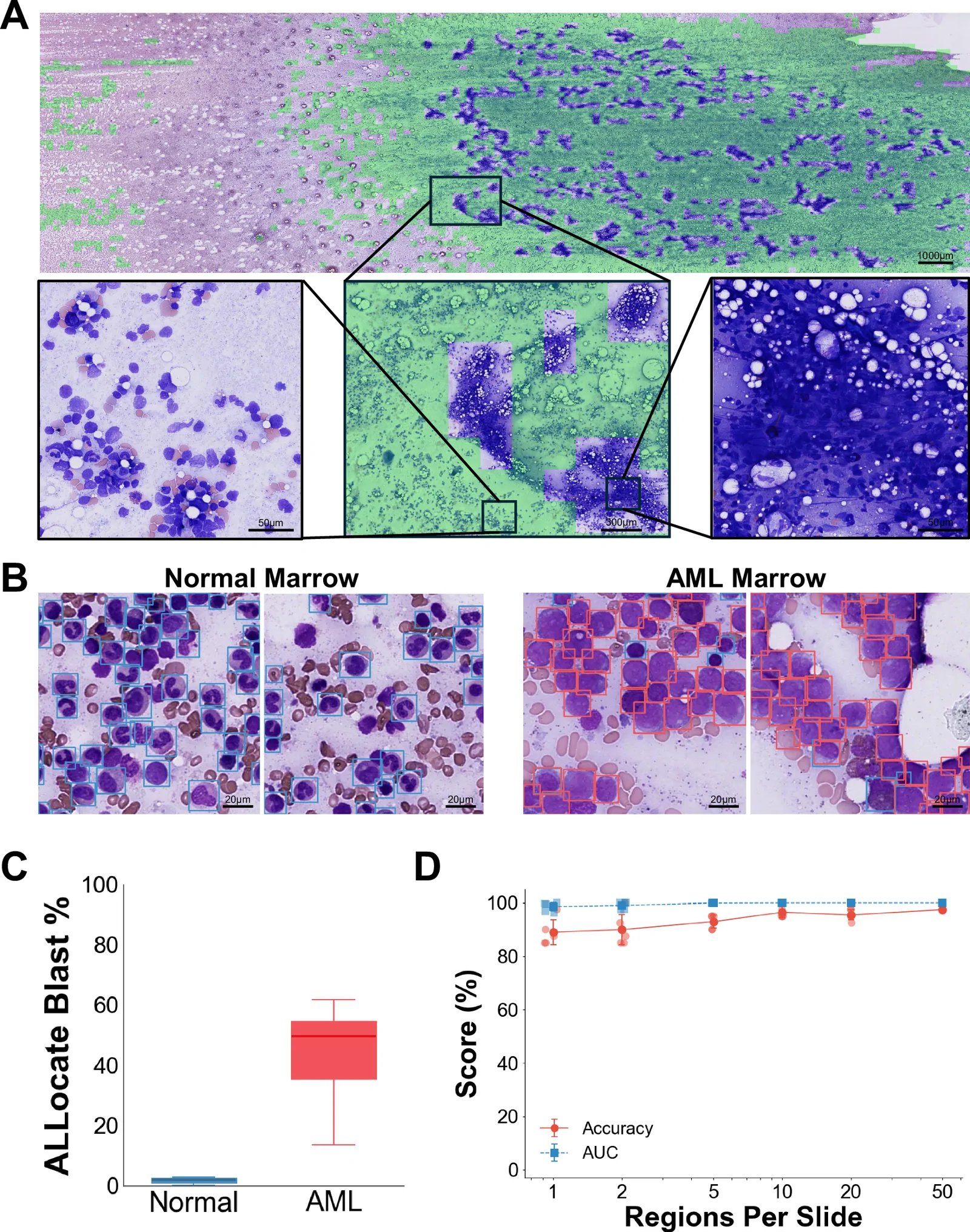
Assessing the model on an external validation cohort on the whole-slide level (EVS). **A** Representative whole-slide image scan from the Memorial Sloan-Kettering Cancer Center and the corresponding region of interest heatmap generated by ALLocate’s region classification system. **B** Sample detections of the model from extracted regions of the whole-slide images. **C** Model prediction representation across normal slides and AML slides. Box plots show the median as the center line, the 25th and 75th percentiles as the lower and upper bounds of the box, and whiskers extending to the most extreme values within 1.5 times the interquartile range (mean blast ratio: 2.0% normal, 44.3% AML). The analysis included 20 normal slides and 20 AML slides (*n* = 40 independent slides from 40 patients), with each slide representing one patient. **D** Model accuracy and AUC score on the MSKCC whole-slide dataset with regions evaluated per slide ranging from 1 to 50, demonstrating the performance of ALLocate with varying sample sizes. Lines represent mean values across five independent folds, and error bands represent ±1 standard deviation (*n* = 5 folds) across the 40-slide cohort. Source data are provided as a Source Data file.

**Fig. 5:**
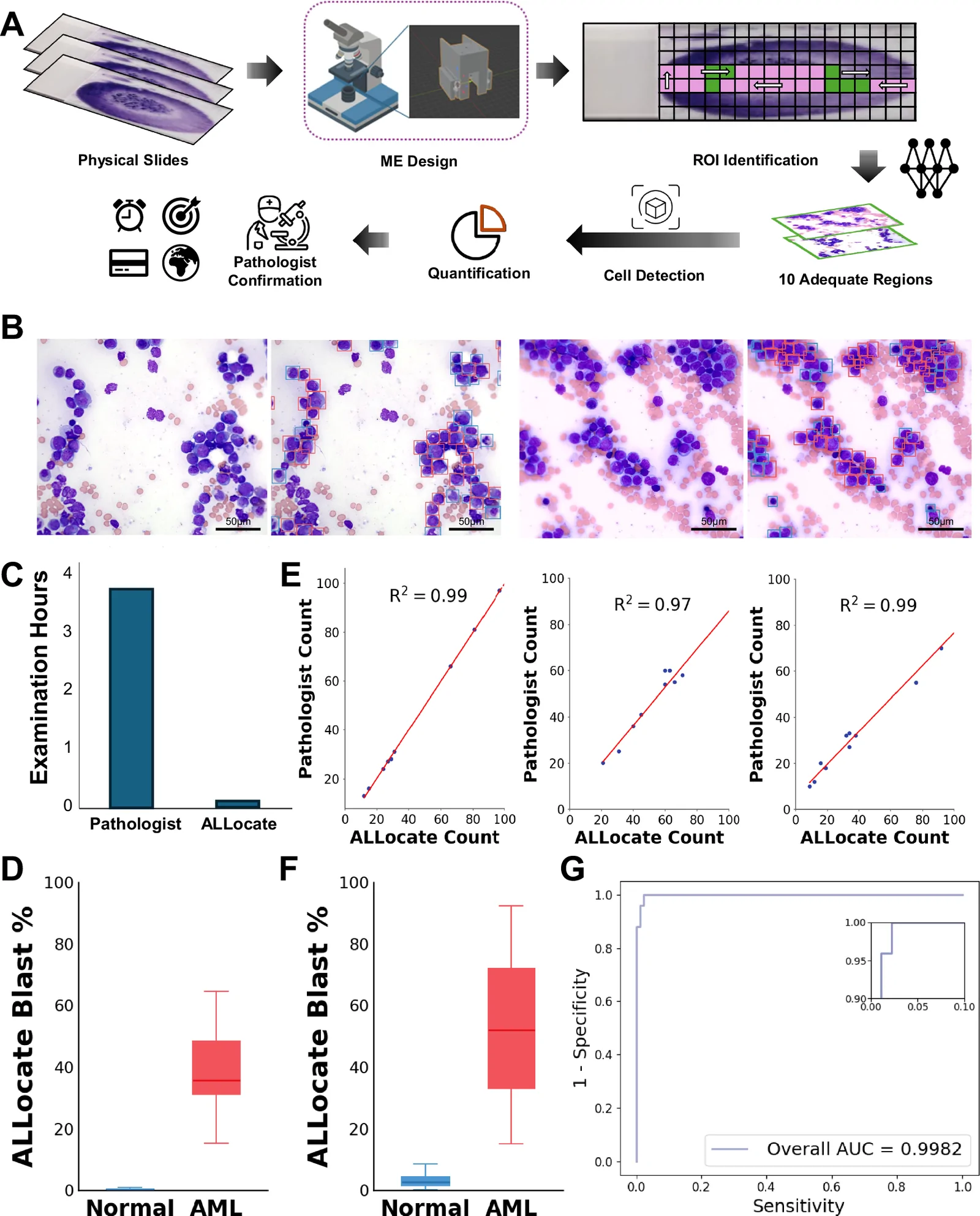
ALLocate achieves superior performance in real-world detection of physical glass slides using the ALLocate microscopic scanner system. **A** Illustration of ALLocate detection logic and process flow for real-world deployment. **B** Two representative smears scanned and detected by ALLocate from the SDM dataset (Blue: normal cells; Red: blasts). **C** Comparison of examination time between pathologists and ALLocate. **D** Overall statistics for blast percentage detection by ALLocate on the UCSF SDM dataset. Box plots show the median as the center line, the 25th and 75th percentiles as the lower and upper bounds of the box, and whiskers extending to the most extreme values within 1.5 times the interquartile range (mean blast ratio: 0.41% normal, 40.28% AML). The UCSF cohort included 18 normal and 18 AML slides (*n* = 36 independent slides from 36 patients), with each slide representing one patient. **E** Sample correlation with linear fit based on ALLocate cell counts (x-axis) and pathologist counts (y-axis) on three representative slides. **F** Overall statistics for blast percentage detection by ALLocate on the external MSKCC SDM dataset. Box plots show the median as the center line, the 25th and 75th percentiles as the lower and upper bounds of the box, and whiskers extending to the most extreme values within 1.5 times the interquartile range (mean blast ratio: 4.04% normal, 51.86% AML). The external MSKCC cohort included 73 normal and 56 AML slides (*n* = 129 independent slides from 129 patients), with each slide representing one patient. **G** ROC curve for slide-level AML classification on the combined UCSF and MSKCC self-driving microscope (SDM) dataset. Ground-truth labels correspond to slide-level diagnoses, and prediction scores are defined by the estimated blast ratios (blast cells / total nucleated cells) produced by ALLocate. Source data are provided as a Source Data file.

Detailed information regarding training is further provided in the Methods section. The overall evaluation framework is detailed in Fig. 1C. It spans multiple levels of analysis, including ROI identification (Fig. 2), blast cell detection (Fig. 3), end-to-end pipeline evaluation on both digital slides (Fig. 4) and physical glass slides acquired using the self-driving microscope system (Fig. 5). In total, the study leverages large-scale annotated datasets at both the region and cell levels, together with multi-institutional external validation cohorts. Table 1 provides a summary of the dataset attributes and tasks we performed.

### ALLocate identifies ROIs with high accuracy

The ALLocate system starts by finding diagnostic regions from digitized slide images. The whole bone marrow aspirate smear contains a variety of complex areas, which can be grouped into three typical categories: clotted areas within the spicules (clot regions), mononuclear cell-enriched areas (adequate regions) and areas with mostly red blood cells (blood regions) (Fig. 2A and Fig. S1). Pathologists routinely scan marrow smears at low magnification to identify mononuclear cell-enriched areas for further cell-level classification. To emulate this process, a CNN-based region classifier was developed to identify suitable regions for further classification, thereby reducing the pipeline's overall processing time and streamlining the detection workflow. The model was trained on 9149 region tiles and evaluated on 2286 region tiles, which were labeled by hematopathologists as either adequate, blood, or clot regions (TR, ER).

Figure 2B shows the model’s performance using ROC curves, with AUC reported for each class. The model demonstrates excellent performance across all region types, with AUCs of 0.992 for adequate regions, 0.998 for blood regions, and 0.997 for clot regions. Validation performance across different hyperparameter settings, as well as detailed evaluation and training logs on a randomly sampled 10% subset of the training data, are provided in Table S2. The high performance (Table S3 and Fig. S2) enables our framework to accurately identify the diagnostic regions prior to downstream blast cell quantification. In addition to numerical performance analysis, a visualization is presented in Fig. 2C to show the reliability of our region classification model, with areas in green representing adequate regions. Overall, the high-performing ROI identification provides a solution for the classification of adequate diagnostic regions as suitable for cell-level analysis.

### Accurate blast cell identification by ALLocate on UCSF and MSKCC cohorts

Given the need to balance accuracy and speed, the YOLOv8 architecture was selected for blast cell detection and classification. The model was trained using hematopathologist-annotated bounding boxes of nucleated cell instances, distinguishing blast cells (indicative of AML in this study) from normal cells (TC, EC; Fig. 3A). Across the dataset, 109,825 cell instances were used for model training (TC), and 26,533 cell instances were used for evaluation (EC). The evaluation metrics used were precision, recall, and mAP50. Detailed training logs on 20% of the training data are provided in Table S4. Overall, our model achieved precision, recall, and mAP50 of 0.84, 0.85, and 0.90, respectively, with consistent performance across cross-validation folds (Fig. 3B).

A comparative analysis between hematopathologists and ALLocate was conducted on a dataset from an external institution (MSKCC). Trained exclusively on UCSF data, the cell detection model was applied to 99 normal regions and 87 AML regions from MSKCC data (EVC). Pathologists also annotated the same set of images. At the region level, ALLocate achieved an AUROC of 0.9012 for distinguishing normal regions from AML-associated regions, compared to 0.9359 for the hematopathologist, using blast ratio as the continuous prediction score (Fig. 3E). Quantitative analysis of blast counts showed that, in normal regions, ALLocate detected a similar number of blasts as the pathologist (mean 0.75 vs. 0.60 blasts per tile; Fig. 3F, Fig. 3G, Table S5, Table S6) without significant statistical difference (*P* = 0.07). In AML regions, the model produced comparable blast counts (mean 10.54, range 0–41) to those of the pathologist (mean 11.13, range 0–37; Fig. 3F, Fig. 3G, Table S5, Table S7), with no statistically significant difference (*P* = 0.12). However, the evaluation time vastly differed, with ALLocate requiring, on average, less than 1 minute for evaluation compared to more than 4 hours for a hematopathologist to manually perform blast counting in 200 regions. Overall, these results demonstrate the effective performance of the local cell-detection model on pre-tiled region samples. Furthermore, Fig. 3C, D provide visual examples showing that ALLocate can successfully distinguish between normal and blast cells in both the UCSF and MSKCC datasets. ALLocate’s high accuracy on both the in-domain (UCSF) and external (MSKCC) test sets underscores its reliability and robustness across platforms.

### AI evaluation: end-to-end validation confirms ALLocate accurately detects leukemia from digitized slides

While validation of individual AI components, including external validation, is essential, it is insufficient to demonstrate this system’s utility as a practical diagnostic tool. To address this, we conducted an end-to-end evaluation of the complete AI pipeline using digitized slides from MSKCC. To assess the performance of the full deep learning system at the slide level in a clinically realistic setting, we curated a testing cohort consisting of 40 digitized bone marrow aspirate slides from MSKCC, comprising 20 normal and 20 AML cases (EVS; Fig. S3). As shown in Fig. 4A, despite being trained exclusively on UCSF data, the model reliably identified diagnostically adequate regions on whole-slide images. With the successful integration of the blast cell detection module (Fig. 4B), the AI pipeline enabled automated slide-level analysis on external samples.

To mimic the human diagnostic workflow, we randomly selected 10 diagnostically adequate regions per slide and performed blast cell counting, yielding a minimum of 200 cells per slide, comparable to manual clinical assessments. All AML slides exhibited higher blast percentages than normal slides, allowing ALLocate to fully distinguish AML from normal cases under an ideal threshold (Fig. 4C and Table S8). To further assess robustness, we varied the number of regions evaluated per slide from 1 (highly underrepresentative) to 50 (highly overrepresentative) (Fig. 4D, Table S9, Table S10). The model achieved near-perfect AUC with as few as 5 regions, demonstrating strong potential as a diagnostic tool. Even when applying a more stringent textbook threshold, which dictates ≤5% blasts for a benign diagnosis and ≥20% for AML, the system achieved 93% accuracy. Together, these results demonstrate the robustness and generalizability of the ALLocate AI algorithm, even when evaluated on a completely external dataset.

### End-to-end evaluation of ALLocate using physical marrow smear slides

To comprehensively evaluate the entire ALLocate pipeline, including both hardware components and AI modules, we processed a total of 165 glass bone marrow smear slides, including 36 glass bone marrow smear slides (SDM, UCSF) from UCSF and an additional 129 glass slides (SDM, MSKCC) from MSKCC through the complete system (Table S11). Moving from the region to the slide level, we applied the diagnostic logic of ALLocate in a manner that mimics the pathologist’s evaluation process (Fig. 5A). ALLocate was installed on a microscope and applied to the physical slides, with its AI component generating blast percentages. Each clinical sample was first digitally captured by the ALLocate hardware. The captured images were then filtered by a region classifier to produce representative images with at least 500 cells for analysis. On AML slides, ALLocate efficiently highlighted blast cells (Fig. 5B and Fig. S4). Compared to the hematopathologist’s evaluation, ALLocate demonstrated strong and consistent performance across both internal and external cohorts. Benign slides exhibited low blast percentages, as confirmed by both AI-based and clinician diagnoses (Fig. 5D, F). The difference in blast percentage between AML and normal samples was highly significant in both the UCSF and MSKCC cohorts (*P* = 4.72 × 10⁻¹⁰ and *P* = 1.77 × 10⁻²³, respectively). In addition to statistical analysis, we applied clinically established thresholds ( ≤ 5% blasts for benign and ≥20% blasts for AML). On the internal UCSF cohort, ALLocate correctly classified 17 out of 18 AML cases (94.4%) and 18 out of 18 normal cases (100%), corresponding to an overall accuracy of 97%. Importantly, performance remained robust on the fully independent external MSKCC cohort, where ALLocate correctly classified 53 out of 56 AML cases (94.6%) and 58 out of 73 normal cases (79.5%), achieving an overall accuracy of 86.0%. This modest decrease in performance on the external cohort is expected due to differences in slide preparation, imaging conditions, and institutional workflows, and further underscores the model’s robustness under real-world variability. Consistent with the separation observed in Fig. 4, the two categories are linearly separable, resulting in an AUC approaching 1.0 (Fig. 5G and Fig. S5). High performance (88% overall accuracy) across the internal (97% accuracy) and external (86% accuracy) SDM datasets highlights the system’s strong diagnostic performance.

For quantitative comparison, pathologists randomly annotated three AML and three normal slides, with 10 diagnostic regions per slide. A strong correlation was observed between the blast counts from ALLocate and the manual annotations across all annotated AML and normal slides, indicating the robustness of ALLocate both overall and at the individual slide level (Fig. 5E and Fig. S6, S7). Overall, on actual physical slides, ALLocate not only excelled in diagnostic accuracy but also achieved pathologist-level performance in quantifying blast counts at a fraction of the time (Fig. 5C).

## Discussion

In this work, we present ALLocate, a study that integrates a deep learning system with a low-cost automated microscope scanner for leukemia detection in whole-marrow smears. We tested ALLocate on different sets of whole marrow smears, including both normal and AML marrows, to show its real-world clinical applicability. To enable cross-institutional generalization, ALLocate was developed using data from the University of California, San Francisco (UCSF) and Memorial Sloan Kettering Cancer Center (MSKCC). The models were trained and validated using UCSF data and then tested on several cohorts of images from MSKCC taken by commercial scanners. Beyond comprehensive software testing, the complete ALLocate pipeline, including the ALLocate Automated Microscope Scanning System, was then applied to 165 physical glass slides, which demonstrated the strong reliability of ALLocate. This intensive testing routine shows ALLocate’s clinical generalizability across multiple hospitals and multiple image capture methods.

A key strength of our approach is its low cost, with a total system cost of less than $150 compared to approximately $300,000 for commercial whole-slide scanners. Together with other emerging low-cost digital microscopy frameworks^23,25^, this work highlights the growing interest in accessible diagnostic imaging platforms. Combined with a CNN-based region classifier and YOLOv8-based cell detection, the proposed system provides an end-to-end solution for automated analysis of bone marrow smears and can be deployed in resource-limited clinical settings. Importantly, both the region classification model and the object detection model are lightweight and can operate entirely on CPU. In cases where high-end hardware is unavailable, the models can be run locally, albeit with slightly longer processing times, or easily executed using cloud-based resources. Beyond cost savings, the system can augment the capacity of overburdened pathologists and hematologists by automating time-consuming tasks such as ROI selection and blast quantification. In regions with few expert personnel, it can serve as a triage tool to prioritize high-risk cases for expert review, or as a training and educational resource for clinicians in diagnostic hematology. By incorporating this design, scanners that cost hundreds of thousands of dollars may not be needed after the successful implementation of this pipeline.

Our approach parallels human pathologists' workflows for bone marrow smear analysis. Whereas the pathologist’s first step is to examine the whole bone marrow smear at low magnification for suitable regions, a CNN-based region classifier breaks a large smear into processing tiles, searching for the most optimal regions. In usable regions, the pathologist examines individual cells and distinguishes between normal and leukemia cells, typically evaluating 200–500 cells to establish the final differential count. Similarly, these qualified regions will be processed by a real-time one-step localization and classification system for leukemia cell detection, mirroring the steps a pathologist would take. The developed region classifier can identify the usable regions in the aspirate smear with an accuracy of 96%, while the YOLOv8 model was developed and optimized to detect marrow cells with a mAP of 90%. The real-time detection using YOLOv8 was also demonstrated in regions as well as whole-slide images. When tested on scanned tiled marrow regions, whole scanned images, and whole marrow slides, ALLocate demonstrated excellent leukemia detection results, but faster than a clinical expert. As an assistive tool, ALLocate can significantly improve the efficiency of leukemia detection from aspirate smears in real-world settings and can potentially be used for clinical screening. Results can be relayed to a qualified expert pathologist for verification, including captured region and cell images.

To contextualize ALLocate’s performance, we compared it with several published deep learning models for bone marrow analysis^12,14–16,19,20,27^ (Table S12). Previous studies have demonstrated impressive achievements using very large datasets. For example, Matek et al. trained a ResNeXt-50 on 171,374 expert-annotated cells from 945 patients, reaching 79% precision for blast detection^12^. Tayebi et al. reported 84% mAP50 using YOLOv4 on data from 1,247 patients^20^, while Lewis et al. built a general differential counting pipeline with 1.5 million cells, achieving 92% mAP50 in AML detection^15^. In comparison, ALLocate achieves 90% mAP50 for blast detection while relying on only one stage of detection, which is computationally more efficient than traditional two-stage systems. It is important to note, however, that while our dataset captures a range of AML subtypes, the sample size within each subtype remains limited, and future studies with larger, subtype-balanced cohorts are needed to fully evaluate model performance across AML variants. In addition, many of these earlier studies addressed the broader and more challenging task of full differential classification, encompassing dozens of bone marrow cell types. While ALLocate focuses specifically on blast detection, these pipelines deserve recognition for the breadth of classes they successfully distinguish. Our work thus complements this body of research by demonstrating that high blast-level accuracy can also be achieved with a streamlined and computationally efficient pipeline.

Although the ALLocate system achieved strong performance, several limitations should be noted. First, while the current hardware enables automated XY stage scanning for slide pre-screening, it lacks automated Z-axis focusing, which, without human input, may result in focal variability across regions. Second, a dedicated graphical user interface for streamlined visualization, case management, and integrated pathologist review has not yet been implemented. Future work will focus on incorporating autofocus functionality and developing a unified interface to facilitate clinical integration. Also, although glass-level validation was performed on both the UCSF dataset (including an independent test set) and the MSKCC external validation cohort, the overall number of physical glass slides remains relatively limited. Larger, multi-center studies will be necessary to further improve statistical power and confirm generalizability across diverse clinical settings. In addition, to our knowledge, there are currently no publicly available bone marrow aspirate smear datasets with blast-level bounding box annotations suitable for object detection tasks. This reflects a broader limitation in the field rather than a constraint specific to this study and highlights the need for standardized, publicly available datasets for benchmarking and future model development. Finally, we observed occasional misclassification of immature granulocytes as blasts, particularly in reactive marrow conditions. These errors most frequently occurred when immature granulocytes exhibited prominent nucleoli, a feature that can overlap morphologically with leukemic blasts and challenge the model’s discriminative capacity. Future work will incorporate a more diverse set of reactive marrow samples to better capture the spectrum of granulocytic maturation and improve model robustness.

Regarding real-world deployment considerations, the algorithm design intentionally reflects a trade-off between sensitivity, precision, and operational speed. The system is intended as a rapid triage and screening tool integrated with an automated microscope, where missed blasts would be clinically more consequential than moderate increases in false positives. Therefore, the detection threshold was tuned to maintain high sensitivity for blast identification while preserving acceptable precision, thereby avoiding an excessive downstream review burden. In practical settings, ALLocate operates as an assistive system rather than a standalone diagnostic replacement: detected regions and cell counts are reviewed in aggregate, and outputs can be verified by a clinician. This design prioritizes rapid identification of potentially malignant regions, efficient slide prescreening, and reduction of manual workload while maintaining clinically meaningful accuracy.

In addition, the current study focuses on distinguishing AML from normal marrow and does not explicitly evaluate performance on other hematologic or metastatic disease categories. For out-of-distribution samples, ALLocate would still generate blast detection outputs and quantitative blast proportion estimates; however, these outputs alone are insufficient for definitive diagnosis and should be interpreted as screening indicators requiring expert review. Furthermore, prediction confidence is inherently provided within the pipeline: the cell-level detector outputs class probabilities, while the blast proportion serves as a continuous, slide-level score that can be used for threshold-based decision-making.

Overall, our study presents a relatively low-cost, end-to-end system for the detection of acute myeloid leukemia, combining two deep learning models with a self-driving microscope controlled by 3D printed adapter hardware. The system costs less than $150, offering a practical alternative to commercial whole-slide scanners priced around $300,000. This affordability, combined with its automation, makes it especially valuable for clinical screening in real-world low-resource settings, where access to advanced imaging infrastructure is limited.

## Methods

### Ethics statement

This study complies with all relevant ethical regulations. The study protocols involving de-identified human bone marrow smear samples were reviewed and approved by the Institutional Review Board of the University of California, San Francisco, and the Institutional Review Board of Memorial Sloan Kettering Cancer Center. The requirement for informed consent was waived because the study used de-identified retrospective samples. The bone marrow smear slides were obtained from archived clinical collections at UCSF and MSKCC. The slides were not destroyed during or after analysis and remain stored at the respective institutions in accordance with institutional policies. Participant sex, gender, and age were not considered in the study design because the study evaluated image-based detection performance using de-identified, retrospectively collected smear slides, and these demographic variables were unavailable across all cohorts. No sex- or gender specific analyses were performed because the model was not designed to predict or evaluate sex- or gender-dependent outcomes.

### Automatic stage system

The ALLocate automated stage was integrated with an Olympus BX40 microscope and Olympus EP50 camera based on a previously described design^26^. For external validation on MSKCC glass slides, images were acquired using an Olympus BX46 microscope equipped with an Olympus DP27 camera and CellSens software.

The system consists of custom 3D-printed components, including motor mounts, couplings, gear assemblies, and linear support structures. Two NEMA 14 stepper motors (1.8° per step, 0.4 A rated current) were used for actuation and controlled via a RAMPS 1.4 shield connected to an Arduino Mega 2560 microcontroller (Fig. 1A). The motors were powered by a 12 V, 10 A supply and driven using A4988 stepper motor drivers configured to match motor voltage requirements. The total hardware cost of the system is approximately $150 (excluding the microscope).

The system leverages the microscope stage's existing manual XY translation knobs for motorized actuation. Custom 3D-printed couplings were designed to interface with the X- and Y-axis control knobs and were secured using a combination of friction-fitting and mechanical fasteners to ensure stable torque transmission. The X-axis motor is directly coupled to the stage control shaft, whereas the Y-axis motion is achieved through a geared coupling due to the coaxial arrangement of the stage controls. Both motors are mounted within a 3D-printed housing that translates with the stage and is supported by steel guide rods to maintain alignment. Automated image acquisition is performed using a unidirectional raster scanning pattern. The stage translates along the X-axis to capture sequential image tiles, followed by an incremental Y-axis shift before scanning the next row in the opposite direction. This alternating scanning sequence enables systematic coverage of the selected slide region and is controlled by a custom Arduino C + + script. The current implementation does not include Z-axis motorization or autofocus functionality, which will be addressed in future work.

### Database

The dataset used for training, validation, and evaluation of the deep learning models was acquired from de-identified bone marrow smear whole-slide images provided by the Department of Laboratory Medicine at the University of California, San Francisco (UCSF)^13^. All slides were scanned at 40× magnification using Aperio AT2 and GT450 scanners without additional Z-axis focusing.

For region classification, a total of 9,149 region tiles were used for training (TR), with an additional 2,286 tiles reserved for evaluation (ER), derived from 70 patient slides (n_slides = n_patients) (Table S13). For cell detection, 109,825 annotated cell instances were used for training (TC), and 26,533 instances were used for evaluation (EC), derived from 170 patient slides (n_slides = n_patients). Model development (training and validation) for both tasks was performed at the region level, as annotations were generated at the tile level and multiple regions were extracted from each slide. The AML cohort included a diverse range of molecular and morphological subtypes encountered in routine clinical practice, including AML with *RUNX1::RUNX1T1* fusion, AML with *CBFB::MYH11* fusion, AML with *KMT2A*-rearrangement, AML with *NPM1*-mutation, AML with *TP53*-mutation, therapy-related AML, AML transformed from myeloproliferative neoplasms, and AML with myelodysplasia-related changes. A detailed subtype distribution is provided in Table S14.

External validation was performed using de-identified datasets from Memorial Sloan Kettering Cancer Center (MSKCC), scanned at 40× equivalent magnification using Hamamatsu S360 and S20 scanners. This included 186 region tiles (99 normal, 87 AML) for partial pipeline evaluation (EVC), derived from 95 patient slides (49 AML, 46 normal), where each slide corresponds to a unique patient (i.e., n_slides = n_patients), and 40 digital slides (20 normal, 20 AML) for full pipeline evaluation (EVS).

To evaluate the end-to-end performance of the integrated hardware-AI system, physical glass slides were tested using the self-driving microscope platform. Internal evaluation at UCSF included 36 slides (18 normal, 18 AML), while external evaluation at MSKCC included 129 slides (73 normal, 56 AML). All slide-level evaluations were performed at the patient level, with each slide corresponding to a unique patient (i.e., n_slides = n_patients), and all external validation cohorts were fully independent and not used during model training. These experiments assessed system performance under realistic clinical workflow conditions, including automated scanning and blast quantification.

### Deep learning model development

This study develops two deep learning models incorporated with ALLocate: a CNN region classifier (Fig. S8) and a YOLOv8 blast cell detector (Fig. S9).

#### Region classification

The CNN region classifier is formulated as a multiclass classification problem. The network was trained using a softmax output layer with cross-entropy loss, which enforces a probability distribution across the three classes (summing to 1). During inference, the predicted class label is obtained using the argmax of the softmax probabilities, and precision-recall metrics were computed based on these discrete class predictions rather than thresholded binary outputs.

The CNN model was implemented using PyTorch^28^, featuring an architecture with one input layer, three convolutional layers with corresponding max pooling layers, and three fully connected layers (Figure S8A). Each convolutional layer uses the rectified linear unit (ReLU) activation function, with the final fully connected layer using a softmax activation function. Prior to training, oversampling was performed on the clot class to ensure balanced data across all region classes. Model optimization was initially performed on a 10% subset of the total training data. A second model architecture was also tested, which included two convolutional and max pooling pairs instead of three. With these architectures, training was conducted with a batch size of 64 images, experimenting with different epochs (5, 7, 10, 20, 50) and alternating learning rates of 0.001 and 0.0005, using the Adam optimizer for stochastic gradient descent (SGD). The final classifier on the full dataset was trained at 10 epochs with a learning rate of 0.0005.

#### Object detection model

Building on the one-step detection and classification advantages of the YOLO model, the YOLOv8 model was chosen for this study (Fig. S9A)^29^. In addition to the normal and blast cell classes, an artifact class was also utilized during training in order to prevent false positive detections of artifacts as normal or blast. The total number of images used for the YOLOv8 model training and validation was 1633. To ensure robust model selection and avoid overfitting, the dataset was first split into a hold-out evaluation set and a training set. A five-fold cross-validation strategy was then applied exclusively within the training set. To achieve an optimal performance in cell detection, the YOLO model was extensively optimized and fine-tuned through multiple configurations of parameters on a 20% subset of the training data. This model was trained with both small and medium default architectures, with the medium configuration featuring additional layers. The learning rate varied from 0.001 to an automatic mode where the model adjusts the rate dynamically. Epochs were tested at 20, 50, 100, and 200, and the model was trained using both the “auto” and SGD optimizers with a batch size of 4. Final training was conducted at 100 epochs with the automatic optimizer.

After training the models, data validation was performed as an initial evaluation of the models’ performance post-training. The CNN and YOLOv8 models were quantitatively assessed on the validation dataset using standard metrics, including accuracy, precision, recall, and F1 score. Precision and recall for the YOLOv8 model were computed on the validation set using standard object-detection evaluation protocols at a predefined confidence threshold (confidence = 0.25), selected based on optimal performance on the validation cohort. Once an object was detected, class assignment was determined by the argmax of class probabilities (blast vs. normal).

Balanced composite metrics were not used because performance was evaluated at the cell level and reported separately for each class. This approach is consistent with object-detection practice and avoids obscuring clinically relevant behavior (e.g., sensitivity to blasts) that could be masked by aggregated or class-balanced metrics. Additional evaluation was performed using mean average precision (mAP50). Loss graphs were generated for all training iterations of the two models, and a precision-recall curve also generated for the YOLOv8 model (Fig. 3B and Fig. S8B, 9B). For the YOLOv8 model, image labeling and preprocessing were performed utilizing the Roboflow image annotation platform, where bounding boxes were manually applied to all cell instances.

### Clinical testing

After development, ALLocate was used for clinical testing. A total of 186 regions (EVC), including 99 from the normal bone marrow region dataset and 87 from an AML region dataset, were sourced from Memorial Sloan-Kettering Cancer Center. This dataset was distinct from the training and validation datasets sourced at UCSF. Normal regions are defined as regions captured from slides of patients without AML, and AML regions are taken from slides of patients with AML. The YOLOv8 object detection model performs inference on individual regions and outputs the detection of the cells and its labels (blast cells vs. normal cells). Region-level ground-truth labels (AML-associated vs. normal) were established by expert hematopathologists. A region was labeled as AML-associated only when the reviewing hematopathologist determined it to contain leukemic features and the assessment was consistent with the corresponding slide-level clinical diagnosis. Regions without leukemic features and concordant with benign slide-level diagnosis were labeled as benign. These curated region-level annotations served as the reference standard for AUROC evaluation.

In addition to training based on regions, a testing cohort of 20 normal and 20 AML whole slide images (EVS) was also used. First, these whole slides were tiled using the OpenSlide Python library. These tiles were then processed through the CNN-based region classification pipeline, which selected 10 regions per case for subsequent analysis by the YOLOv8 detection framework; the final model differential counts were evaluated on a case-by-case basis. For each slide, the performance of the model was compared to the reported pathologist’s review, and accuracy designations were decided using the clinical thresholds of ≤5% blasts for normal cases and ≥20% blasts for leukemia cases.

Moreover, another testing cohort of 91 normal and 74 AML whole-slides images was collected using the AAMSS (SDM), including 18 normal and 18 AML bone marrow smear slides from UCSF and 73 normal and 56 AML marrow smear slides from MSKCC, as opposed to the slides scanned by commercial hardware in the previous test. The images captured were then processed through the CNN region classification pipeline and subsequently processed by the YOLOv8 detection framework; the final model differential counts were evaluated on a case-by-case basis. For each slide, the performance of the model was compared to the reported pathologist’s review, using the clinical thresholds of ≤5% blasts for normal cases and ≥20% blasts for leukemia cases.

### Computational hardware

Model training and primary validation were performed on a desktop workstation equipped with an AMD Ryzen 9 7900X CPU, NVIDIA GeForce RTX 4070 Ti SUPER GPU, and 32 GB RAM (Windows 11), utilizing GPU acceleration.

External validation and evaluation of the automated microscopy system were conducted on a laptop computer with an Intel Core Ultra 185H CPU, NVIDIA RTX 4070 Laptop GPU, and 16 GB RAM (Windows 11), also using GPU acceleration.

### Statistical analysis and evaluation metrics

Statistical analyses were performed to evaluate agreement between ALLocate and pathologist assessments, as well as overall model performance. Differences in blast counts between ALLocate and pathologist evaluations on the same regions were assessed using two-tailed paired t-tests. Differences in blast percentage between AML and normal slides were assessed using two-tailed independent t-tests. Correlation analysis was performed to quantify agreement between automated and expert-derived blast counts.

Classification performance was evaluated using receiver operating characteristic (ROC) curve analysis, with area under the curve (AUC) reported. For cell detection tasks, standard object detection metrics were used, including precision, recall, and mean average precision at an intersection-over-union threshold of 0.5 (mAP50). All statistical analyses were performed using standard scientific computing libraries. A *p*-value of <0.05 was considered statistically significant where applicable.

### Reporting summary

Further information on research design is available in the Nature Portfolio Reporting Summary linked to this article.

## Supplementary information

Supplementary Information

Description of Additional Supplementary Files

Supplementary Data 1

Reporting Summary

Transparent Peer Review file

## Source data

Source Data

## Data Availability

A de-identified subset of the ALLocate dataset, comprising 20 annotated leukemia-associated and normal bone marrow region images from the University of California, San Francisco, is publicly available at https://doi.org/10.5281/zenodo.21895819. The complete digitized datasets used for model training and evaluation are not publicly available because they contain clinical imaging data subject to patient privacy, ethical, and institutional data-governance restrictions. Investigators at academic or non-profit institutions may request access for non-commercial research by contacting the corresponding author. Requests must describe the proposed research, the specific data requested, and the intended use. Access is subject to institutional approval, applicable ethical and privacy requirements, and execution of a data-use agreement. The physical glass slides are not available for distribution because they are human biospecimens retained by the originating institutions. Source data are provided with this paper.

## Code availability

The code used to develop and evaluate the ALLocate system is available at the public GitHub repository (https://github.com/goldgoflab/ALLocate) and has been permanently archived on Zenodo at https://doi.org/10.5281/zenodo.21879578, under the Apache License, Version 2.0^30^.
