# Supplementary material for: An AI-powered self-driving microscope for low-cost acute leukemia detection
Source: Nat Commun. 2026 Aug 27;17:10304. doi: 10.1038/s41467-026-77130-x (PMC13619560; doi:10.1038/s41467-026-77130-x)
Supplement: Supplementary file 1 [file 41467_2026_77130_MOESM1_ESM.pdf]

Supplementary Information

Table S1. Detailed Summary of Datasets Used in the Study

Dataset Purpose		Inst.	Hardware	Data Type	Qty	Details
TR	Training	UCSF	40x/400x-equivalent WSI images (Aperio AT2, GT450)	Region Tiles	9,149	Train the region classifier to identify diagnostically relevant regions.
TC		UCSF	40x/400x-equivalent WSI images (Aperio AT2, GT450)	Cell Instances	109,825	Train the cell detector to locate nucleated cells and classify blast cells.
ER	Evaluation	UCSF	40x/400x-equivalent WSI images (Aperio AT2, GT450)	Region Tiles	2,286	Evaluate the region classifier's ability to identify diagnostic regions.
EC		UCSF	40x/400x-equivalent WSI images (Aperio AT2, GT450)	Cell Instances	26,533	Evaluate the cell detector's performance on nucleated cell localization and blast classification.
EVC	External Validation	MSKCC	40x/400x-equivalent WSI images (Hamamatsu S360, S20)	Region Tiles	186 (99 Normal, 87 AML)	Evaluate partial pipeline performance on external data; compare cancerous region detection with pathologist annotations.
EVS		MSKCC	40x/400x-equivalent WSI images (Hamamatsu S360, S20)	Digital Slides	40 (20 Normal, 20 AML)	Evaluate the full AI pipeline on digitized slides from the external institution.
SDM	Self Driving Microscope	UCSF	40x/400x self-driving compound microscope (Olympus BX40, 3D printed actuators, Arduino controller)	Glass Slides	36 (18 Normal, 18 AML)	Test the complete hardware + AI system on physical slides to simulate real-world clinical workflow.
		MSKCC	40x/400x self-driving compound microscope (Olympus BX46, 3D printed actuators, Arduino controller)	Glass Slides	129 (73 Normal, 56 AML)	Test the complete hardware + AI system on physical slides from an external institution to simulate real-world clinical workflow.

Table S2. Region Classifier Development Using Convolutional Neural Network

**# of Epochs**	5	5	5	5	7	10	20	20	50
**Architecture**	3 Conv. Layers, 3 Max Pooling Layers	2 Conv. Layers, 2 Max Pooling Layers	3 Conv. Layers, 3 Max Pooling Layers	3 Conv. Layers, 3 Max Pooling Layers	3 Conv. Layers, 3 Max Pooling Layers	3 Conv. Layers, 3 Max Pooling Layers	3 Conv. Layers, 3 Max Pooling Layers	3 Conv. Layers, 3 Max Pooling Layers	3 Conv. Layers, 3 Max Pooling Layers
**Learning Rate**	0.0001	0.0005	0.0005	0.001	0.0005	0.0005	0.0005	0.001	0.0005
**Regularization**	None	None	None	None	None	None	0.005	0.005	0.005
**Overall Accuracy**<br>Adequate<br>Blood<br>Clot	0.89<br>0.78<br>0.98<br>0.94	0.95<br>0.91<br>0.98<br>0.97	0.96<br>0.93<br>0.98<br>0.94	0.90<br>0.77<br>0.99<br>0.97	0.95<br>0.91<br>0.99<br>0.89	0.88<br>0.73<br>0.99<br>0.95	0.95<br>0.93<br>0.98<br>0.92	0.94<br>0.88<br>0.98<br>0.97	0.93<br>0.86<br>0.98<br>0.97
**Overall Precision**<br>Adequate<br>Blood<br>Clot	0.86<br>0.97<br>0.90<br>0.72	0.92<br>0.98<br>0.99<br>0.78	0.92<br>0.96<br>0.100<br>0.81	0.95<br>0.92<br>0.89<br>0.97	0.93<br>0.97<br>0.95<br>0.87	0.83<br>0.99<br>0.96<br>0.56	0.93<br>0.96<br>0.97<br>0.87	0.89<br>0.98<br>0.88<br>0.71	0.89<br>0.98<br>0.98<br>0.70
**Overall Recall**<br>Adequate<br>Blood<br>Clot	0.90<br>0.77<br>0.98<br>0.94	0.95<br>0.91<br>0.98<br>0.97	0.95<br>0.93<br>0.98<br>0.94	0.93<br>0.87<br>0.99<br>0.92	0.92<br>0.91<br>0.99<br>0.89	0.89<br>0.73<br>0.99<br>0.94	0.94<br>0.93<br>0.98<br>0.92	0.94<br>0.88<br>0.98<br>0.97	0.92<br>0.86<br>0.98<br>0.97
**Overall F1 Score**<br>Adequate<br>Blood<br>Clot	0.87<br>0.86<br>0.94<br>0.82	0.93<br>0.94<br>0.99<br>0.86	0.94<br>0.95<br>0.99<br>0.87	0.92<br>0.89<br>0.94<br>0.94	0.93<br>0.94<br>0.97<br>0.88	0.84<br>0.84<br>0.98<br>0.70	0.94<br>0.95<br>0.98<br>0.89	0.91<br>0.93<br>0.99<br>0.82	0.90<br>0.91<br>0.98<br>0.81

Table S3. Detailed validation metrics (Precision, Recall, F1) for the region classification model

	**Overall**	**Adequate**	**Blood**	**Clot**
**Precision**	0.95	0.95	0.97	0.94
**Recall**	0.96	0.93	0.98	0.96
**F1 Score**	0.95	0.94	0.97	0.95

Table S4. Summary of validation performance on data subset using the ALLocate cell detection model

**# of Epochs**	20	20	50	50	100	100	100	200	200	300
**Learning Rate**	auto	0.001	auto	0.001	0.001	auto	auto	auto	auto	0.001
**YOLOv8 Version**	Small	Small	Small	Small	Small	Small	Medium	Small	Medium	Medium
**Overall Precision**<br>Normal<br>Blast	0.88<br>0.95<br>0.80	0.90<br>0.87<br>0.92	0.86<br>0.91<br>0.81	0.92<br>0.95<br>0.98	0.80<br>0.79<br>0.80	0.83<br>0.83<br>0.83	0.83<br>0.82<br>0.83	0.84<br>0.88<br>0.79	0.83<br>0.88<br>0.77	0.82<br>0.80<br>0.83
**Overall Recall**<br>Normal<br>Blast	0.62<br>0.40<br>0.83	0.12<br>0.03<br>0.21	0.72<br>0.49<br>0.94	0.24<br>0.08<br>0.39	0.81<br>0.71<br>0.90	0.85<br>0.84<br>0.85	0.83<br>0.84<br>0.81	0.73<br>0.55<br>0.91	0.76<br>0.60<br>0.92	0.79<br>0.73<br>0.85
**Overall mAP**<br>Normal<br>Blast	0.76<br>0.68<br>0.83	0.51<br>0.45<br>0.57	0.77<br>0.70<br>0.84	0.58<br>0.63<br>0.52	0.85<br>0.80<br>0.90	0.91<br>0.91<br>0.90	0.89<br>0.89<br>0.88	0.81<br>0.71<br>0.91	0.82<br>0.90<br>0.74	0.83<br>0.78<br>0.87

Table S5. Statistics from ALLocate blast count on the MSKCC external cell-level validation cohort (EVC)

	Normal	AML
Average	0.75	10.54
Min	0	0
Max	6	41
Standard Dev.	1.20	9.36

Table S6. Statistics from ALLocate blast count in normal regions compared with hematopathologist on the MSKCC external cell-level validation cohort (EVC)

	ALLocate	Pathologist
Average	0.75	0.60
Min	0	0
Max	6	5
Standard Dev.	1.20	0.90

Table S7. Statistics from ALLocate blast count in AML regions compared with hematopathologist on the MSKCC external cell-level validation cohort (EVC)

	ALLocate	Pathologist
Average	10.54	11.13
Min	0	0
Max	41	37
Standard Dev.	9.36	8.24

Table S8. Blast percentages predicted by ALLocate on the MSKCC external slide-level validation cohort (EVS)

Normal	AML
0%	35%
2%	53%
1%	50%
3%	55%
2%	14%
3%	52%
1%	45%
2%	17%
2%	54%
2%	34%
1%	36%
2%	45%
0%	56%
1%	56%
1%	37%

7%	62%
2%	22%
0%	61%
2%	50%
3%	53%

Table S9. Accuracy for predictions by ALLocate on the MSKCC external slide-level validation cohort (EVS) sample simulation across five bootstrap iterations

Regions	Bootstrap Iteration				
	1	2	3	4	5
1	0.85	0.98	0.88	0.85	0.90
2	0.93	1.00	0.85	0.85	0.88
5	0.95	0.95	0.90	0.95	0.90
10	0.95	1.00	0.95	0.98	0.95
20	0.95	0.98	0.93	0.98	0.95
50	0.98	0.98	0.98	0.98	0.98

Table S10. AUC for predictions by ALLocate on the MSKCC external slide-level validation cohort (EVS) sample simulation across five bootstrap iterations

Regions	Bootstrap Iteration				
	1	2	3	4	5
1	1.00	1.00	0.96	1.00	0.97
2	1.00	1.00	0.98	0.98	1.00
5	1.00	1.00	1.00	1.00	1.00
10	1.00	1.00	1.00	1.00	1.00
20	1.00	1.00	1.00	1.00	1.00
50	1.00	1.00	1.00	1.00	1.00

Table S11. Breakdown of case-level datasets used in this study

Task/Dataset Description	Institution	Number of cases
Region classification	UCSF	70
Cell detection	UCSF	170
Region + cell detection (external evaluation)	MSKCC	95
Digital slides (external evaluation)	MSKCC	40
Physical slides (internal evaluation)	UCSF	36
Physical slides (external evaluation)	MSKCC	129

Table S12. Comparison of the deep learning systems in this study with previous literature

**Reference**	**Chandradevan et al. 2020**	**Matek et al. 2021**	**Tayebi et al. 2022**	**Lewis et al. 2022**	**Nakamura et al. 2022**	**Lv et al. 2023**	**Wang et al. 2023**	**Current Study 2025**
Whole Slide Processing	Yes	No	Yes	Yes	Yes	Yes	Yes	Yes
Region Classification	None	N/A	DenseNet	CNN	None	Image Processing	None	CNN
Single Network Detection	No	Yes	Yes	No	No	Yes	No	Yes
ML Algorithms	Faster R-CNN VGG16	ResNeXt-50	YOLOv4	Fast R-CNN CNN	U-Net, Semi-Supervised	CNN	YOLOX-s MLFL-Net	YOLOv8
Performance	Detect AUC-PvR 0.96 Classification ROC-AUC 0.98	Blasts: Prec. 0.79 Rec. 0.69 F1 0.74	Blasts: Prec. 0.87 Rec. 0.90 F1 0.88 mAP50 0.84	Benign mAP50 0.87 AML mAP50 0.92	Classify: Acc. 0.98 Rec. 0.98 Rec. 0.98 F1 0.98	PPV 0.76 NPV 0.99 Sensitivity 0.80 Specificity 0.99	Detect AUC-PvR 0.98 Classify Acc. 0.90	Prec. 0.84 Rec. 0.85 F1 0.84 mAP50 0.90
External Clinic Validation	No	Yes	No	No	No	Yes	No	Yes
Slide Level Predictions	No	No	No	No	No	No	Yes	Yes
Glass Slide Processing	No	No	No	No	No	No	No	Yes

Table S13. Class distribution and prevalence in training and test datasets

**Class**	**Train**	**Test**	**Total**	**Prevalence (%)**
Adequate	3,386	846	4,232	37.01%
Blood	3,386	846	4,232	37.01%
Clot	2,377	594	2,971	25.98%
**Total**	9,149	2,286	11,435	100%

Table S14. Subtypes of AML in the cell annotation dataset

**Type**	**Prevalence**
AML with *RUNX1::RUNX1T1* fusion	6
AML with *CBFB::MYH11* fusion	2
AML with *KMT2A* rearrangement	6
AML with *MECOM* rearrangement	3
AML with *NUP98* rearrangement	1
AML with *NPM1* mutation	7
AML with *CEBPA* mutation	1
AML, myelodysplasia-related	12
AML with *TP53* mutation	5
Therapy-related AML	5
AML transformed from MPN	3
AML with germline *DDX41* mutation	1
AML with *FLT3*	5
AML with *IDH2*	1
AML, NOS	12

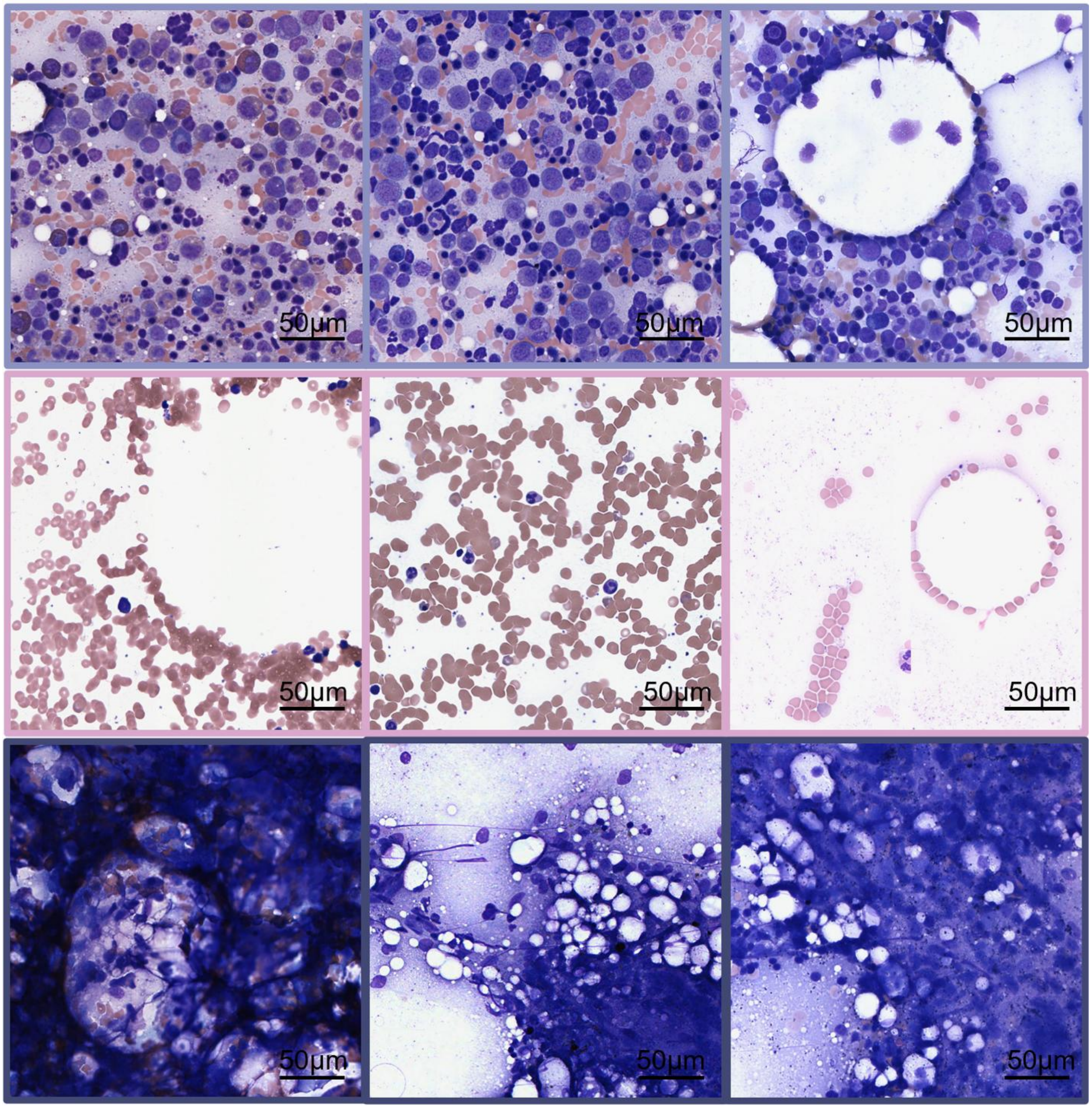

Figure S1. Examples from the three region classes used for region classification. From top row to bottom row: adequate, blood, clot.

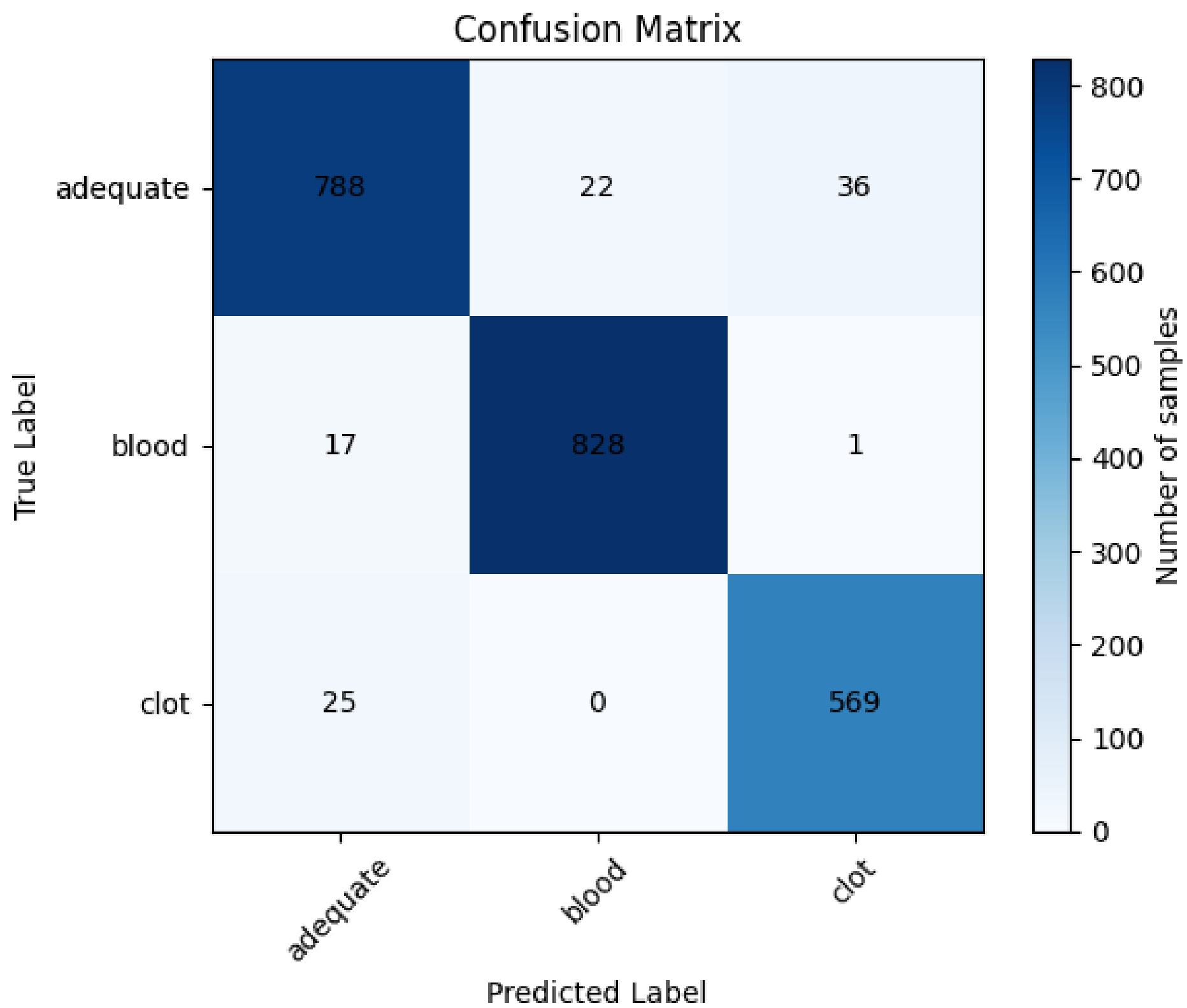

Figure S2. Confusion matrix of region classifier performance.

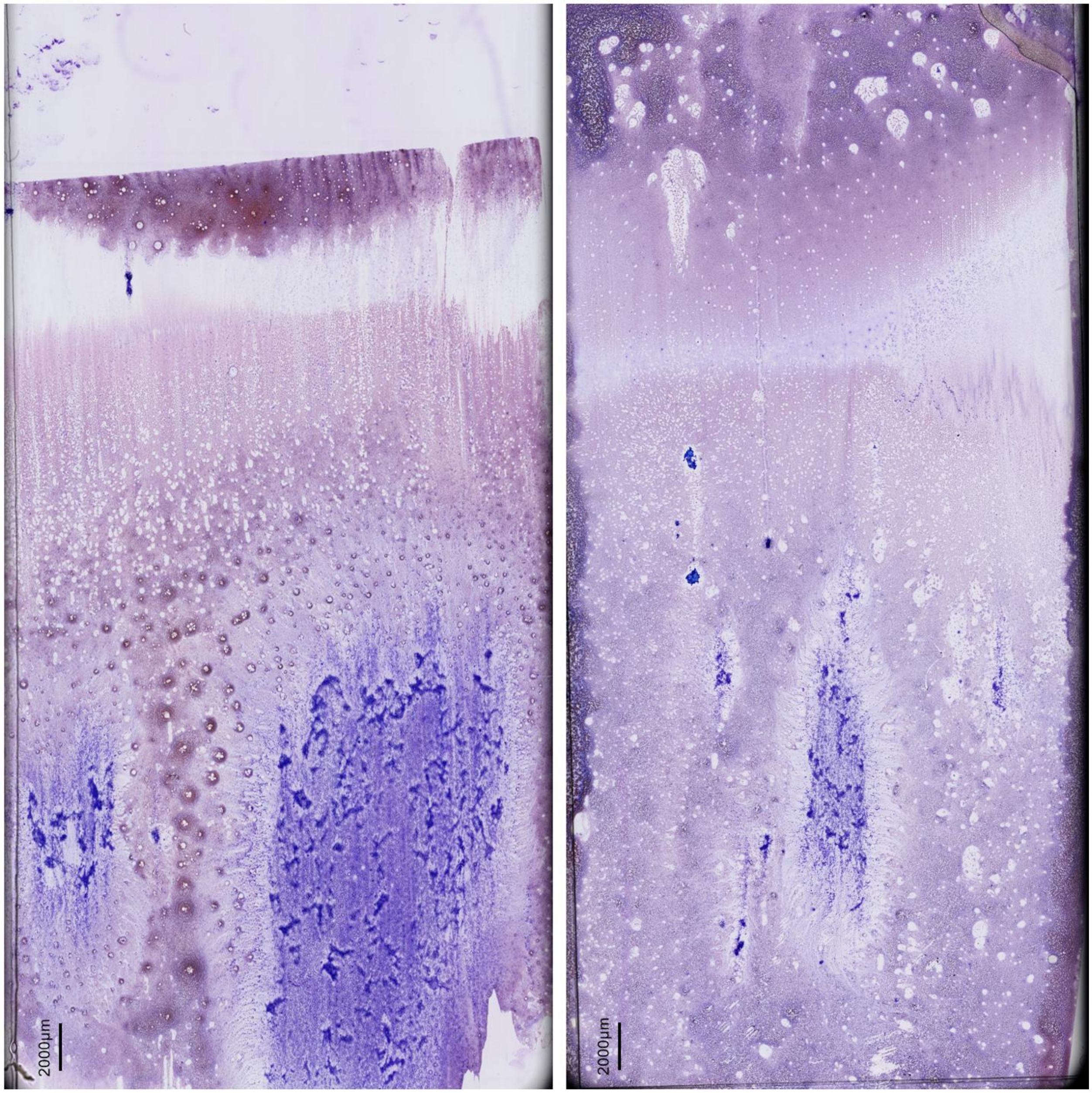

Figure S3. Examples of scanned whole-slide images from the MSKCC external slide-level validation cohort (EVS).

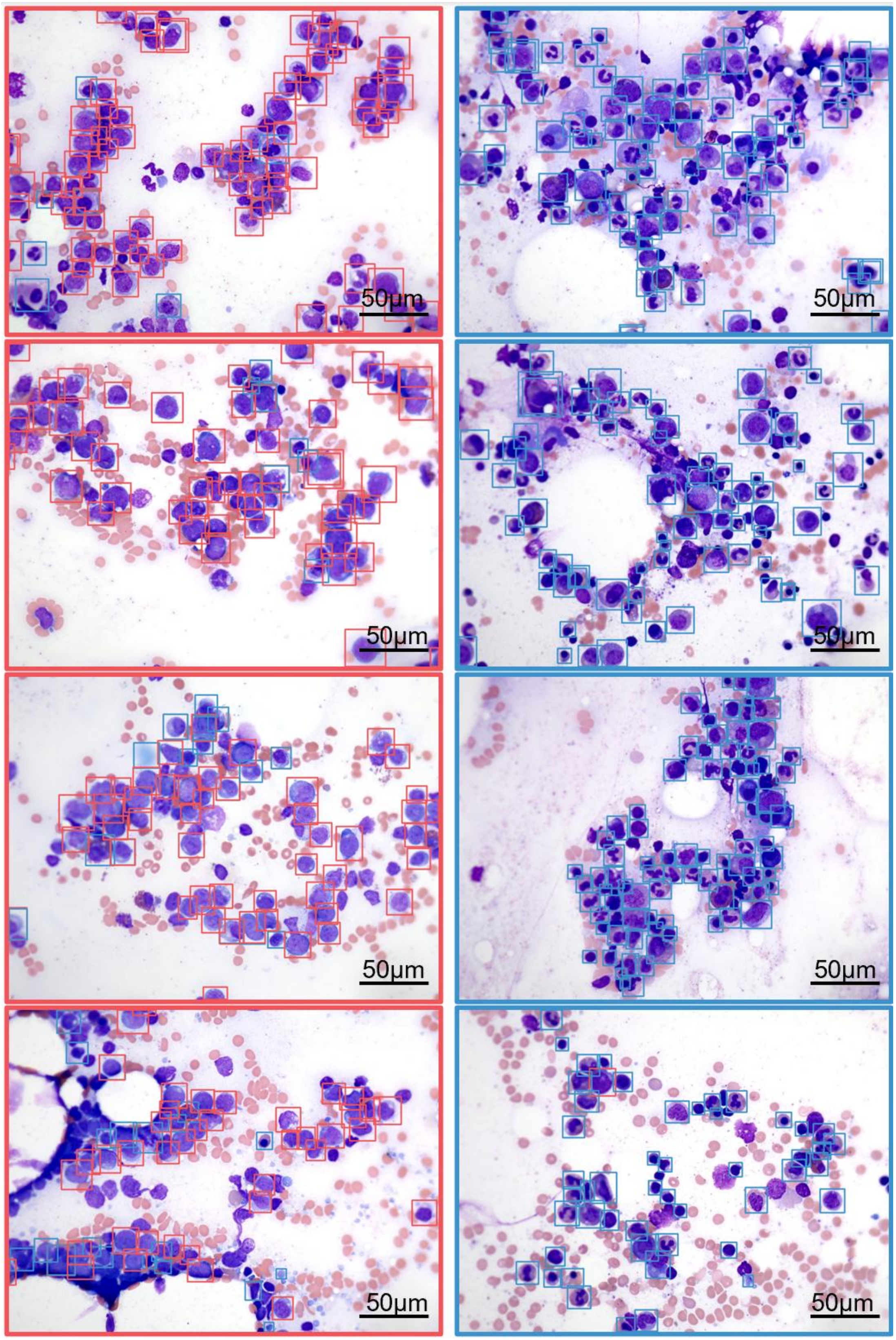

Figure S4: Further examples of ALLocate predictions on the glass slide dataset (SDM).

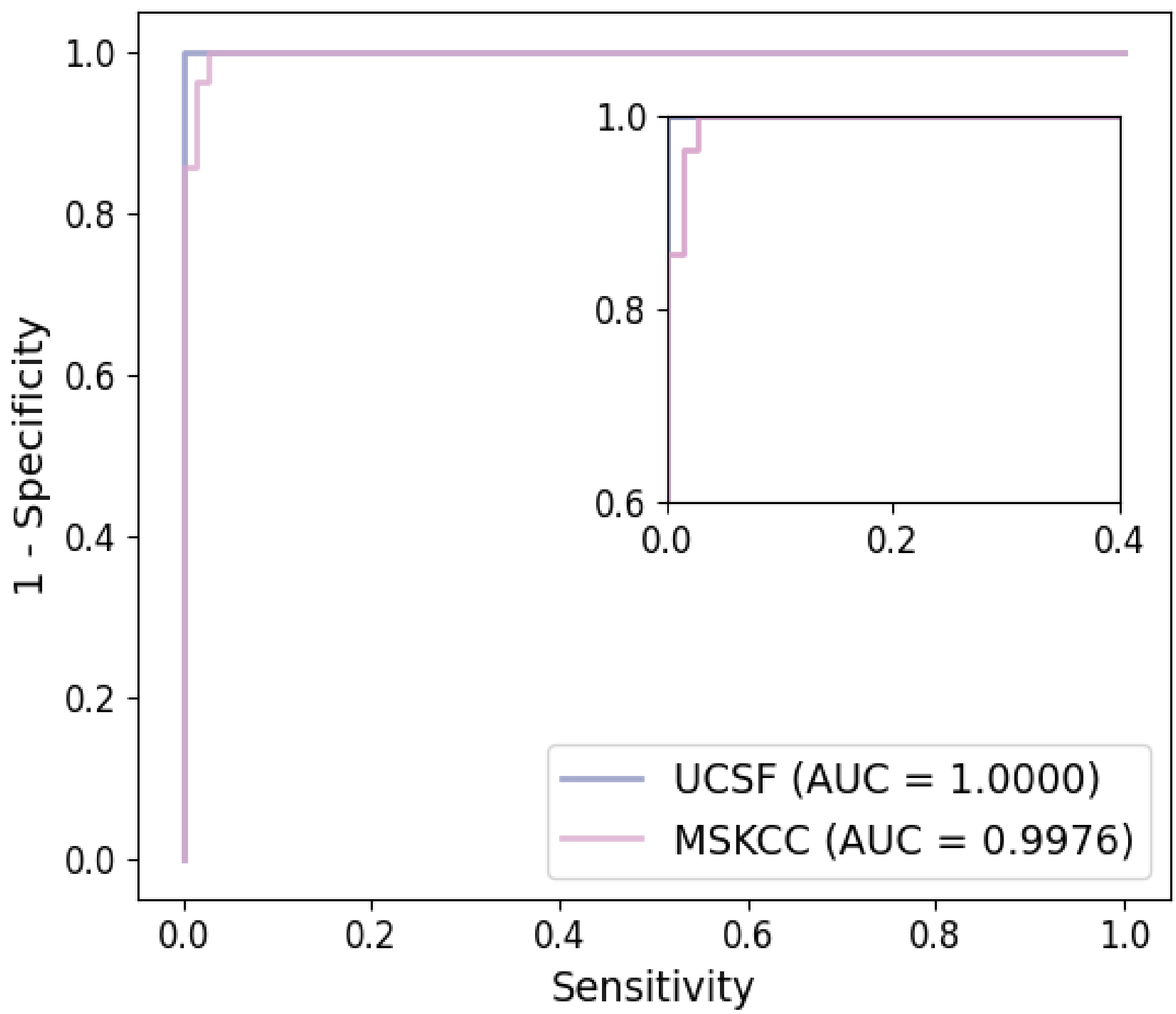

Figure S5. ROC curves for the UCSF and MSK SDM dataset detections by ALLocate.

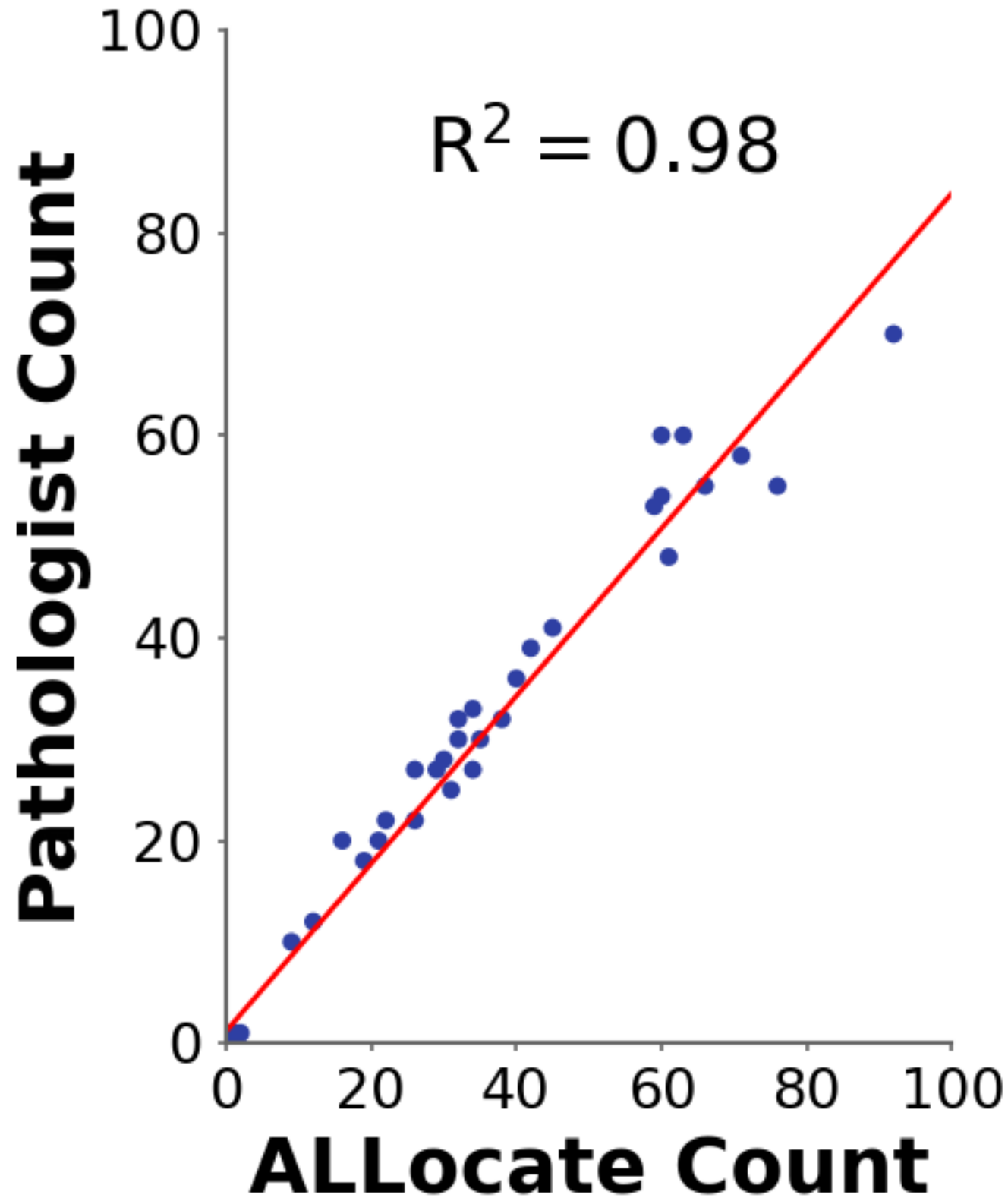

Figure S6. Sample correlation with linear fit based on ALLocate cell counts (x-axis) and pathologist counts (y-axis) on 6 representative slides (3 normal, 3 AML) from the SDM dataset. Individual points represent region-level counts within a slide.

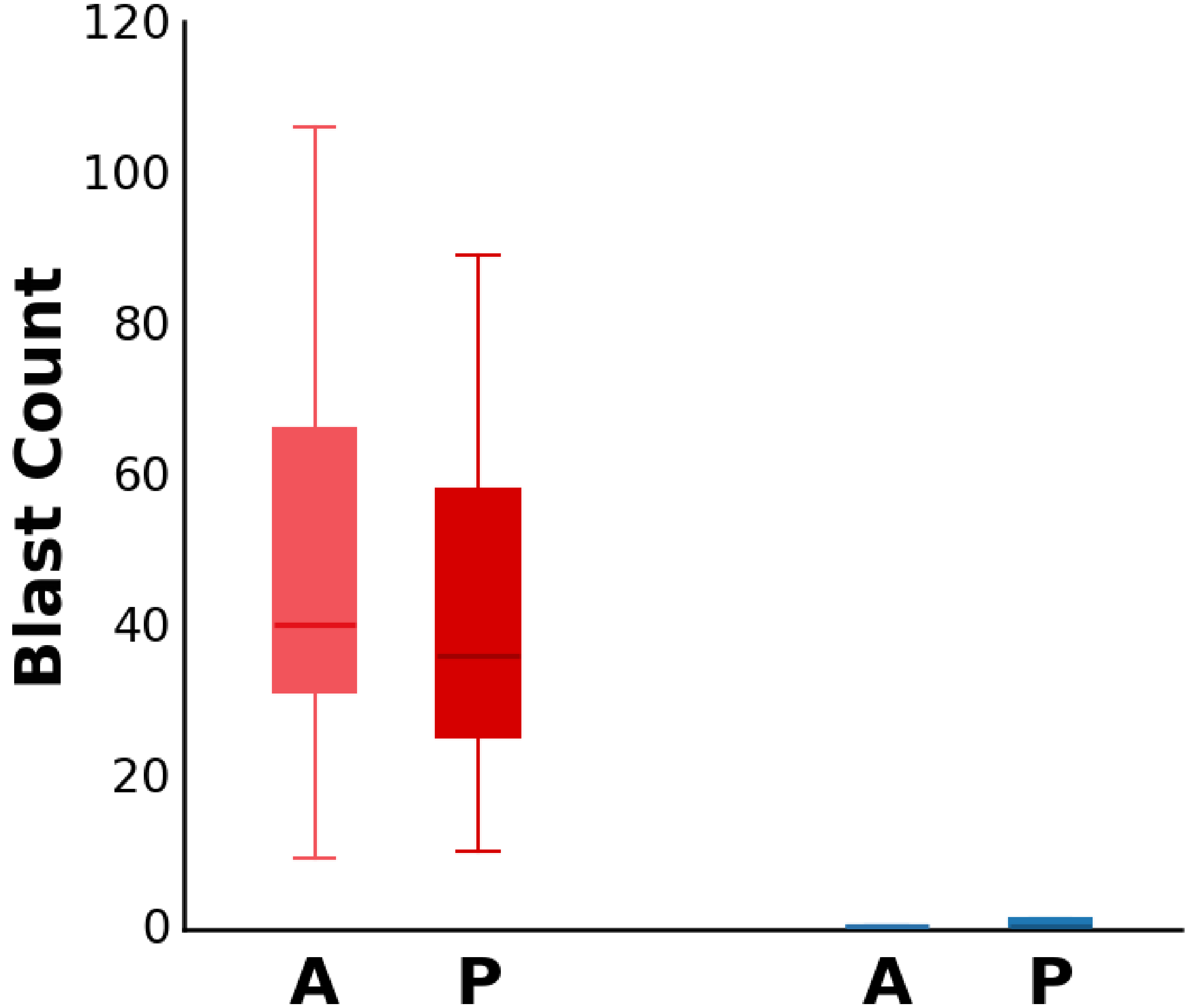

Figure S7. Numerical blast count statistics by ALLocate and pathologists on a six-slide subset (3 normal, 3 AML) of the SDM dataset for AML (red) and benign (blue) slides (A: ALLocate, P: Pathologists). Box plots show the median as the centre line, the 25th and 75th percentiles as the lower and upper bounds of the box, and whiskers extending to the most extreme values within 1.5 times the interquartile range (mean: 49.1 AML/A, 42.4 AML/P, 0.2 Normal/A, 0.4 Normal/P).

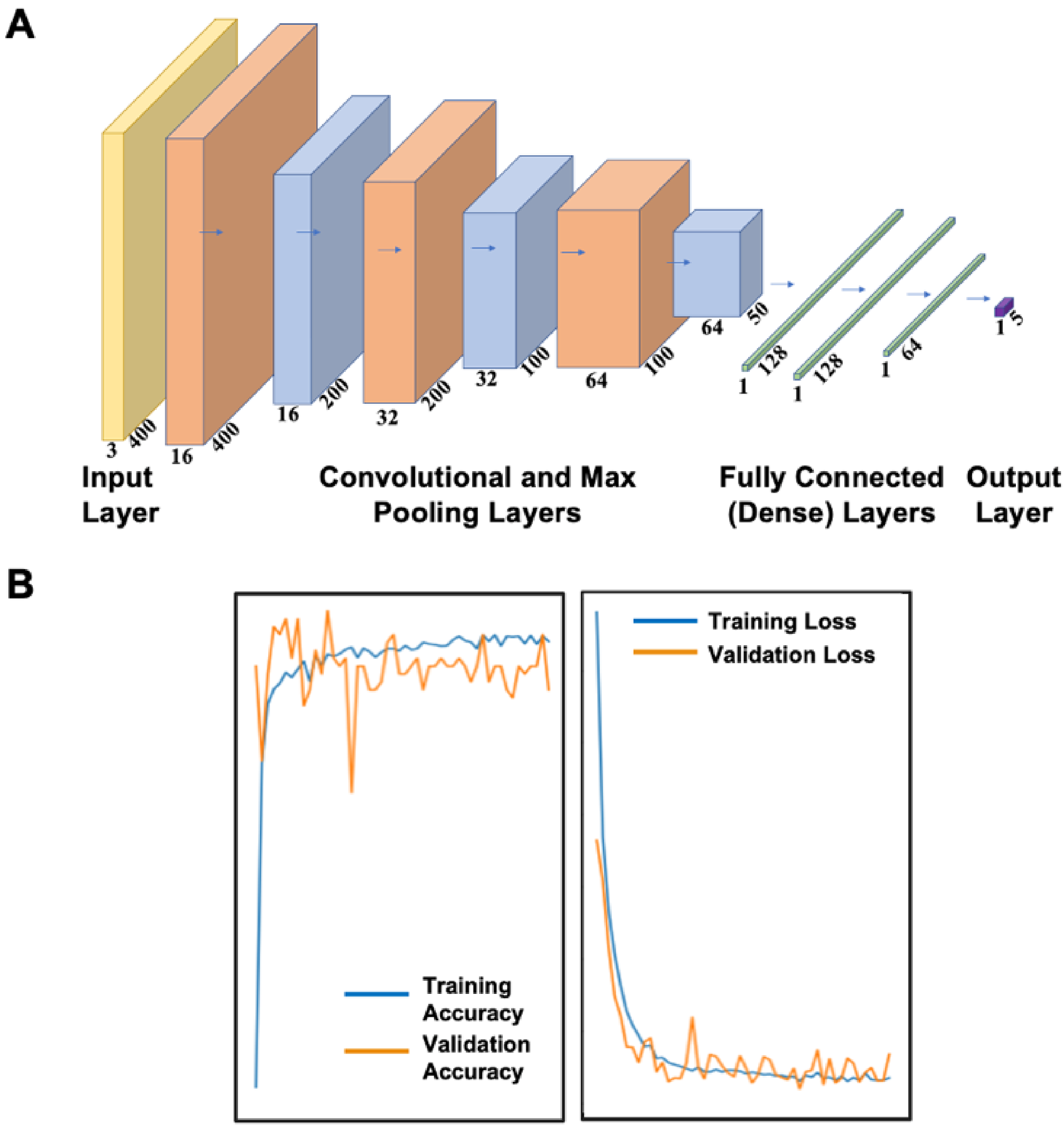

Figure S8. A. CNN model architecture used to create the region classification system.
B. Loss curve of region classifier model.

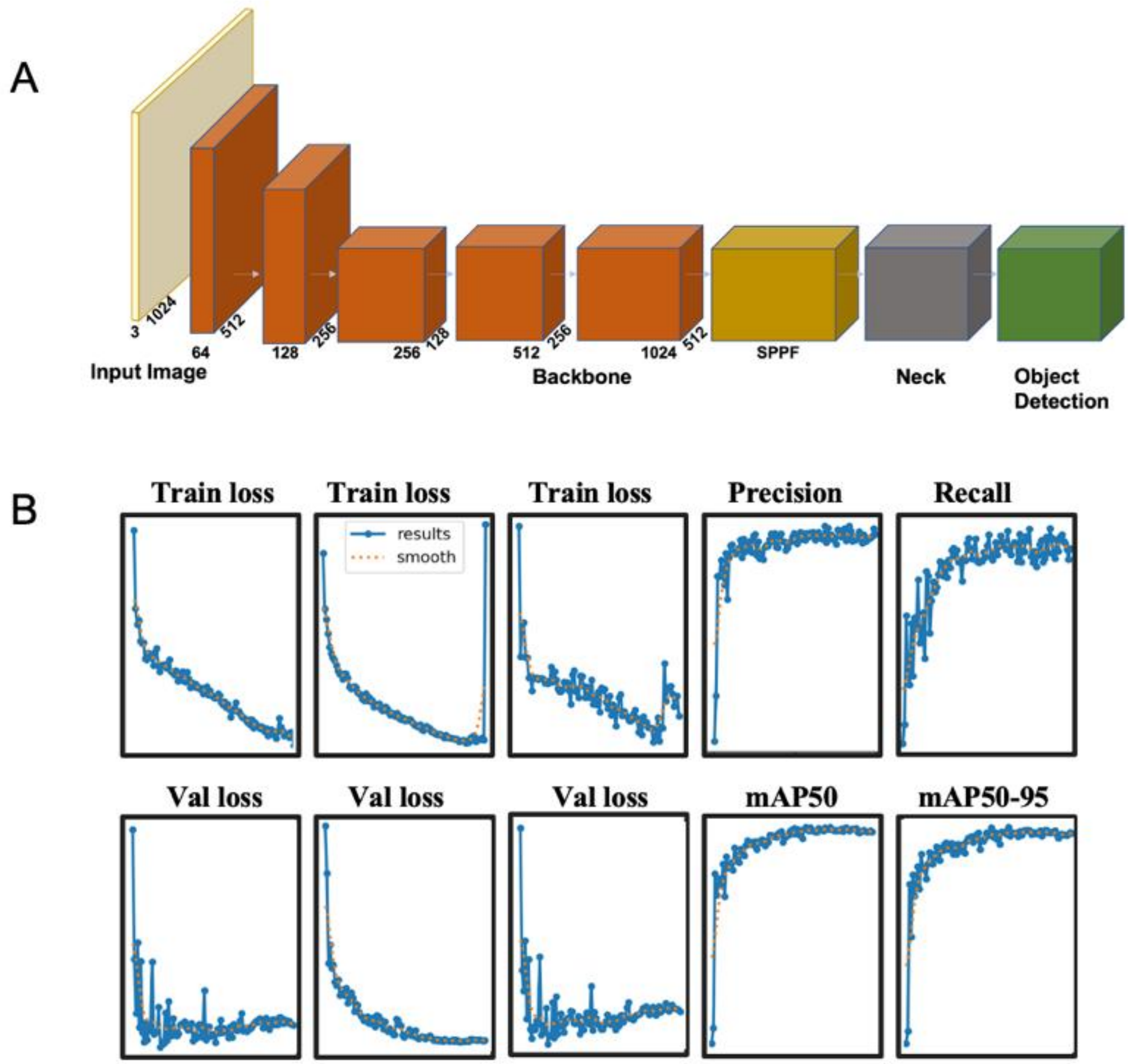

Figure S9. A. YOLOv8 model architecture used to create the ALLocate cell detection system. B. Training logs of the ALLocate cell detection model.
