# Supplementary material for: An AI-powered self-driving microscope for low-cost acute leukemia detection
Source: Nat Commun. 2026 Aug 27;17:10304. doi: 10.1038/s41467-026-77130-x (PMC13619560; doi:10.1038/s41467-026-77130-x)
Supplement: Supplementary file 2 [file 41467_2026_77130_MOESM2_ESM.pdf]

Description of Additional Supplementary Files

Supplementary Data 1: Customized 3D-print design files for the ALLocate microscope-stage hardware, submitted separately to support reproducibility of the hardware system.
