# Supplementary material for: An AI-powered self-driving microscope for low-cost acute leukemia detection
Source: Nat Commun. 2026 Aug 27;17:10304. doi: 10.1038/s41467-026-77130-x (PMC13619560; doi:10.1038/s41467-026-77130-x)
Supplement: Supplementary file 3 [file 41467_2026_77130_MOESM4_ESM.pdf]

nature portfolio

Corresponding author(s): Gregory M. Goldgof

Last updated by author(s): August 11, 2026

Reporting Summary

Nature Portfolio wishes to improve the reproducibility of the work that we publish. This form provides structure for consistency and transparency in reporting. For further information on Nature Portfolio policies, see our Editorial Policies and the Editorial Policy Checklist.

Statistics

For all statistical analyses, confirm that the following items are present in the figure legend, table legend, main text, or Methods section.

n/a	Confirmed	
☐	☒	The exact sample size (n) for each experimental group/condition, given as a discrete number and unit of measurement
☐	☒	A statement on whether measurements were taken from distinct samples or whether the same sample was measured repeatedly
☐	☒	The statistical test(s) used AND whether they are one- or two-sided<br>*Only common tests should be described solely by name; describe more complex techniques in the Methods section.*
☒	☐	A description of all covariates tested
☒	☐	A description of any assumptions or corrections, such as tests of normality and adjustment for multiple comparisons
☐	☒	A full description of the statistical parameters including central tendency (e.g. means) or other basic estimates (e.g. regression coefficient) AND variation (e.g. standard deviation) or associated estimates of uncertainty (e.g. confidence intervals)
☒	☐	For null hypothesis testing, the test statistic (e.g. F, t, r) with confidence intervals, effect sizes, degrees of freedom and P value noted<br>*Give P values as exact values whenever suitable.*
☒	☐	For Bayesian analysis, information on the choice of priors and Markov chain Monte Carlo settings
☒	☐	For hierarchical and complex designs, identification of the appropriate level for tests and full reporting of outcomes
☒	☐	Estimates of effect sizes (e.g. Cohen's d, Pearson's r), indicating how they were calculated

Our web collection on statistics for biologists contains articles on many of the points above.

Software and code

Policy information about availability of computer code

Data collection	Custom Arduino C++ control scripts were used to operate the ALLocate Automated Microscope Scanning System (AAMSS) via the RepRap Arduino Mega Polulu Shield (RAMPS 1.4) and Arduino Mega 2560 controller. The hardware control and image capture relied on open-source firmware compatible with the Arduino IDE (version 1.8.19). No commercial software was used for data collection.
Data analysis	Image preprocessing, tiling, model development, statistical analysis, and figure generation were performed using custom Python scripts. The software and packages used in this study included PyTorch 2.7.1+cu128, Ultralytics YOLOv8 (2023 release), OpenSlide Python 1.4.1, NumPy, OpenCV, Pillow, pandas, scikit-learn, matplotlib, seaborn, Albumentations, and TensorBoard. Data annotation was performed using the Roboflow web platform (https://app.roboflow.com). The custom analysis scripts, model configurations, and compatible package requirements are available in the public GitHub repository (https://github.com/goldgoflab/ALLocate) and have also been permanently archived on Zenodo at DOI: 10.5281/zenodo.21879578, under the Apache License, Version 2.0.

For manuscripts utilizing custom algorithms or software that are central to the research but not yet described in published literature, software must be made available to editors and reviewers. We strongly encourage code deposition in a community repository (e.g. GitHub). See the Nature Portfolio guidelines for submitting code & software for further information.

Data

Policy information about availability of data

All manuscripts must include a data availability statement. This statement should provide the following information, where applicable:

- Accession codes, unique identifiers, or web links for publicly available datasets
- A description of any restrictions on data availability
- For clinical datasets or third party data, please ensure that the statement adheres to our policy

A de-identified subset of the ALLocate dataset, comprising 20 annotated leukemia-associated and normal bone marrow region images from the University of California, San Francisco, is publicly available at DOI: 10.5281/zenodo.21895819. The complete digitized datasets used for model training and evaluation are not publicly available because they contain clinical imaging data subject to patient privacy, ethical, and institutional data-governance restrictions. Investigators at academic or non-profit institutions may request access for non-commercial research by contacting the corresponding author. Requests must describe the proposed research, the specific data requested, and the intended use. Access is subject to institutional approval, applicable ethical and privacy requirements, and execution of a data-use agreement. The physical glass slides are not available for distribution because they are human biospecimens retained by the originating institutions. Source data are provided with this paper.

Research involving human participants, their data, or biological material

Policy information about studies with human participants or human data. See also policy information about sex, gender (identity/presentation), and sexual orientation and race, ethnicity and racism.

Reporting on sex and gender	Participant sex, gender, and age were not considered in the study design because the study evaluated image-based detection performance using de-identified, retrospectively collected smear slides, and these demographic variables were not available for all cohorts. No sex- or gender specific analyses were performed because the model was not designed to predict or evaluate sex- or gender-dependent outcomes.
Reporting on race, ethnicity, or other socially relevant groupings	Not applicable
Population characteristics	De-identified bone marrow smear samples from adult patients at UCSF and Memorial Sloan Kettering Cancer Center were used; no additional demographic or clinical covariates were included.
Recruitment	No direct participant recruitment occurred. Archival clinical specimens were used under institutional approvals with all identifiers removed prior to analysis.
Ethics oversight	This study was conducted in accordance with institutional guidelines and was approved by the Institutional Review Boards of Memorial Sloan Kettering Cancer Center (IRB #16-1591) and the University of California, San Francisco (IRB #2442610).

Note that full information on the approval of the study protocol must also be provided in the manuscript.

Field-specific reporting

Please select the one below that is the best fit for your research. If you are not sure, read the appropriate sections before making your selection.

☒ Life sciences ☐ Behavioural & social sciences ☐ Ecological, evolutionary & environmental sciences

For a reference copy of the document with all sections, see nature.com/documents/nr-reporting-summary-flat.pdf

Life sciences study design

All studies must disclose on these points even when the disclosure is negative.

Sample size	Sample sizes were determined based on the availability of de-identified bone-marrow smear data from institutional archives rather than statistical power calculations. The study included 9,149 region tiles and 109,825 annotated cell instances for training, 2,286 region tiles and 26,533 cell instances for internal evaluation, 186 region tiles (99 normal, 87 AML) and 40 digitized slides (20 normal, 20 AML) for external machine learning validation from MSKCC, 36 physical glass slides (18 normal, 18 AML) from UCSF plus 129 physical glass slides (73 normal, 56 AML) for real-world testing using the self-driving microscope. These sample sizes provided sufficient diversity to train, validate, and externally test model robustness across institutions and imaging modalities.
Data exclusions	No data were excluded other than low-quality or unreadable images identified during preprocessing (e.g., focus or staining artifacts), following pre-established image quality criteria.
Replication	The study consists of computational analyses rather than laboratory experiments. Model performance was assessed using independent training, validation, and test datasets, including external validation cohorts from a separate institution. The YOLOv8 model was additionally evaluated using five-fold cross-validation. Where applicable, results were reproduced across independent cohorts and data partitions rather than through repeated biological experiments.

Randomization	Randomization was not applicable, as this study used pre-existing de-identified clinical specimens rather than experimental interventions. Training and validation splits were randomly assigned within the UCSF dataset to prevent bias.
Blinding	Pathologists labeling the data were blinded to AI predictions during annotation and evaluation. Model performance assessment was performed independently from the data annotation process. Blinding was not applicable during model training because supervised learning requires access to the reference labels. During model evaluation, predictions were generated computationally without manual adjustment based on the clinical diagnosis. Investigators performing the computational analysis did not use group allocation to influence model outputs. Clinicians involved in case identification, data access, and reference-label verification necessarily had access to the corresponding slide and diagnostic information. The model predictions were compared with the reference diagnoses only after inference was completed.

Reporting for specific materials, systems and methods

We require information from authors about some types of materials, experimental systems and methods used in many studies. Here, indicate whether each material, system or method listed is relevant to your study. If you are not sure if a list item applies to your research, read the appropriate section before selecting a response.

Materials & experimental systems

n/a	Involved in the study
☒	☐ Antibodies
☒	☐ Eukaryotic cell lines
☒	☐ Palaeontology and archaeology
☒	☐ Animals and other organisms
☐	☒ Clinical data
☒	☐ Dual use research of concern
☒	☐ Plants

Methods

n/a	Involved in the study
☒	☐ ChIP-seq
☒	☐ Flow cytometry
☒	☐ MRI-based neuroimaging

Clinical data

Policy information about clinical studies

All manuscripts should comply with the ICMJE guidelines for publication of clinical research and a completed CONSORT checklist must be included with all submissions.

Clinical trial registration	Not applicable. This study did not involve a clinical trial or prospective human participants.
Study protocol	Not applicable. The study used retrospective, de-identified bone marrow smear images under institutional review and did not require a clinical protocol or registration.
Data collection	De-identified bone marrow smear slides were obtained from the pathology archives of the University of California, San Francisco (UCSF) and Memorial Sloan Kettering Cancer Center (MSKCC). Slides were originally prepared during routine clinical diagnostics between 2019 and 2024, scanned using Aperio or Hamamatsu scanners, or imaged using the ALLocate self-driving microscope system.
Outcomes	The primary outcome was the diagnostic accuracy of the ALLocate AI system for distinguishing acute myeloid leukemia (AML) from benign marrow based on smear images; secondary outcomes included region-of-interest identification performance and blast quantification accuracy compared with pathologist counts.

Plants

Seed stocks	*Report on the source of all seed stocks or other plant material used. If applicable, state the seed stock centre and catalogue number. If plant specimens were collected from the field, describe the collection location, date and sampling procedures.*
Novel plant genotypes	*Describe the methods by which all novel plant genotypes were produced. This includes those generated by transgenic approaches, gene editing, chemical/radiation-based mutagenesis and hybridization. For transgenic lines, describe the transformation method, the number of independent lines analyzed and the generation upon which experiments were performed. For gene-edited lines, describe the editor used, the endogenous sequence targeted for editing, the targeting guide RNA sequence (if applicable) and how the editor was applied.*
Authentication	*Describe any authentication procedures for each seed stock used or novel genotype generated. Describe any experiments used to assess the effect of a mutation and, where applicable, how potential secondary effects (e.g. second site T-DNA insertions, mosiacism, off-target gene editing) were examined.*
