# Supplementary material for: An AI-powered self-driving microscope for low-cost acute leukemia detection
Source: Nat Commun. 2026 Aug 27;17:10304. doi: 10.1038/s41467-026-77130-x (PMC13619560; doi:10.1038/s41467-026-77130-x)
Supplement: Supplementary file 4 [file 41467_2026_77130_MOESM5_ESM.pdf]

nature portfolio

Peer Review File

An AI-Powered Self-Driving Microscope for Low-Cost Acute Leukemia Detection

Corresponding Author: Professor Gregory Goldgof

This file contains all reviewer reports in order by version, followed by all author rebuttals in order by version.

Attachments originally included by the reviewers as part of their assessment can be found at the end of this file.

Version 0:

Reviewer comments:

Reviewer #1

(Remarks to the Author)
In this study, Yan and Sun et al developed ALLocate, an affordable, AI-powered microscope workflow that enables accurate and efficient leukemia detection. The effective design of a CNN-based region classifier followed by a YOLOv8-based cell detection and classification frees up the pipeline from the need of whole-slide images. The authors designed comprehensive evaluation tasks and demonstrated impressive model performance at every stage of the pipeline. Overall, it is an innovative tool with great potentials for leukemia detection in a resource-limited real-world setting.

My minor comments fall into the following 3 categories:
- The results are convincing, but some technical details in model evaluations are missing. Adding those could further strengthen the paper, help readers who are not familiar with some assumptions in the leukemia world, and make sure the work can be reproduced, e.g.
- Assuming the CNN region classifier should be a multiclass model, rather than a multilabel model. It would be nice to explicitly mention that. Therefore it will be clear that the precision recall was computed using the predicted class (argmax) rather than a pre-defined binarization threshold. It would be helpful to provide the prevalence of the 3 classes, and confusion matrix to help understand the model behavior.
- For YOLOv8 performance evaluation, figure 3B, how were the precision and recall determined? Using a balanced metric? Can you include some discuss around how the trade-off in real-world application factor into the algorithm design here?
- It is unclear how the pathologist performance (auroc) was derived from Figure 3E. Is there a reference pathologist for 'ground truth' annotation, and an evaluation pathologist? Is number of blast used here for classification?
- The authors need to pay more attention to the figure legends and ensure the panel descriptions match the figures, and the information in the figure is self-sufficient, e.g.
- Figure 1A, it would be helpful to add (1)-(5) in the figure to help reader understand the hardware setup. The ALLocate price in the panel ($100) does not align with the main text ($150)
- Figure 5, the figure legend for the panels do not match the content - one extra panel and the order is incorrect
- More effective visualizations could be used to highlight the message, e.g.
- Figure 3B bar plot with error bars - side note: confidence intervals should be consistently reported especially given the very high model performance
- Figure 3G - it is unclear whether paired samples are used in the analysis, then paired visualization should be used, e.g. swarmplot with connected dots or scatter plot

(Remarks on code availability)
The code is pretty readable and seems to contain the bulk of the results. However, dependencies are needed to set up the repo and run the code.

Reviewer #2

(Remarks to the Author)
This article proposes an approach aimed at democratizing AI-based automatic leukemia detection on whole bone marrow smears through a low-cost, AI-powered automatic microscope scanner called ALLocate. The AI module combines a CNN

region classifer with a YOLOv8-based leukemia detector. Training and validation were conducted on a private monocentric dataset of 55 digitalized whole marrow smears, while testing was perfomed on 40 external digitalized slides. Finally, 36 monocentric marrow smear slides were used for combined hardware+ AI component evaluation (i.e. End-to end evaluation). ALLocate achieved an AUC >0.99 for region-of-interest detection, a mean average precision (mAP50) >0.9 for cell detection and an overall diagnostic accuracy of 89% at the glass slide level.

AI-based algorithms for cancer detection and classification, including leukemia detection on blood/marrow smears, have already been published with remarkable performance. However, major challenges such as limited generalizability and poor explainability remain major obstacles to their clinical adoption. In my opinion, this work does not adequately address these issues and remains essentially another proof of concept study with significant limitations (particularly the very small size of the datasets) and limited novelty, apart from the implementation of a low-cost microscope scanning system.

Major comments :
1. Limited dataset size and heterogeneity:
The number of cases used for training, validation, and testing is unclear and appears surprisingly limited for a CNN-based machine learning approach. AML is a heterogeneous group with multiple differentiation and molecular subtypes leading to significant morphological variability. This heterogeneity is not discussed in this paper (which AML subtypes were used?) and cannot be reflected in these limited datasets.
2. Limited diagnostic scope and lack of prediction confidence:
The dataset includes only AML and normal marrow smears. This binary classification task is overly simplistic and does not reflect real diagnostic practice, where cytologists and pathologists must differentiate among a broad spectrum of diseases (e.g., acute lymphoblastic leukemia, myelodysplasia, chronic MPNs, lymphomas, metastases). What prediction would ALLocate generate on these out of distribution input data ? (meaning non-AML and non-normal samples).
Moreover, the system provides no confidence index for its predictions, making it difficult to assess their reliability.
3. Need to clarify the methodology :
The number of cases used is unclear. It would be better to specify the number of WSIs and the number of patients rather than referring to “regions”. Particularly, it would be relevant to precise the number of WSI and patients included in the different datasets in Table 1.
For ROI and cell detection tasks, the methods does not specify whether the train/test split was performed at the patch, slide, or patient level (the latter being the most appropriate to avoid data leakage).
The authors do not share their code or comment on it potential availability after publication.

4. Lack of external validation for the self driving microscope task :
The final end-to-end evaluation of ALLocate was performed only on a limited number (n=36) of internal, monocentric slides. Validation on external cohorts, preferably from multiple centers, is essential to demonstrate the robustness of ALLocate. In fact, interlaboratory variability can significantly affect the generalizability of such AI systems, especially when trained on a monocentric dataset (which is the case here).
Moreover, validation of the AI module on public datasets would also help reinforce its generalizability.

Minor comments :
1. Figure panels and legends require correction.
2. Does ALLocate also work on peripheral blood smears ?
3. Specify which statistical tests were used.

(Remarks on code availability)
The authors do not share their code or comment on it potential availability after publication.

Reviewer #3

(Remarks to the Author)
Summary and noteworthy results
This manuscript reports the development and validation of ALLocate, a low-cost, AI-enabled self-driving microscope system for automated detection of acute myeloid leukemia (AML) from bone marrow smear slides. The most noteworthy results are:
1. The successful integration of inexpensive, 3D-printed motorized hardware with a two-stage machine-learning pipeline.
2. Demonstration of robust performance across internal datasets, external institutional datasets, and—most importantly—physical glass slides, which is rarely shown in comparable studies.
3. Competitive blast detection accuracy (mAP ≈ 0.91) and slide-level diagnostic accuracy (~89–92%) without reliance on whole-slide scanners.
The end-to-end validation on real slides is a particularly strong and impactful aspect of the work.

Significance and relationship to existing literature
The work is highly relevant and significant to the fields of computational pathology, digital hematopathology, and global health diagnostics. It addresses a major limitation of much existing literature: the dependence on expensive whole-slide imaging infrastructure.
Relative to established literature, the manuscript:
• Compares favorably with prior deep-learning approaches for bone marrow analysis in terms of blast detection performance.
• Is distinct in its system-level contribution, combining low-cost hardware, AI inference, and real-world validation.
However, while the manuscript situates the AI performance well within prior hematopathology literature, it would benefit from a broader discussion of edge-based, low-cost, or open-source microscopy systems, which are increasingly relevant to the

stated deployment goals.

Support for conclusions and claims
Overall, the data presented support the main conclusions that ALLocate can accurately identify diagnostically relevant regions, detect blast cells, and distinguish AML from benign cases using a low-cost automated microscope. The use of external validation datasets strengthens these claims.
That said, some conclusions related to deployability and clinical readiness would be further strengthened by:
• Clearer description of system architecture (camera, computing platform).
• Explicit clarification of current limitations, such as lack of autofocus and absence of a human-in-the-loop review interface.
These do not undermine the main claims but should be clearly framed as current limitations.

Data analysis, interpretation, and conclusions
The data analysis is generally sound and appropriate for the study goals. Evaluation metrics (AUC, mAP, precision/recall, accuracy) are standard and well chosen. Interpretation of results is mostly balanced.
No major analytical flaws are evident. However:
• The relatively small number of physical glass slides should be discussed more explicitly in terms of statistical power.
• Failure modes (e.g., misclassification of immature granulocytes) are acknowledged but could be better illustrated.
These issues warrant revision and clarification, not rejection.

Methodology and technical soundness
The methodology is technically sound and meets expected standards for the field. The staged ML approach (ROI selection followed by cell detection) is appropriate and clinically motivated.
However, several aspects of the hardware and acquisition methodology require clearer description:
• The stage actuation mechanism is described largely as a list of components, without sufficient mechanistic detail.
• Firmware logic and tile acquisition strategy are only described at a high level.
• Z-axis focusing and autofocus are not implemented; this limitation should be stated explicitly in the Methods, not only in the Discussion.

Reproducibility and methodological detail
At present, the manuscript does not provide enough detail for full reproducibility of the hardware system. Specifically:
• Camera specifications and image acquisition control are not described.
• The computing platform used for image capture and ML inference is not explicitly stated.
• Mechanical coupling, motion resolution, and scan logic of the automated stage are insufficiently detailed.
Providing additional schematics, flow diagrams, or expanded textual descriptions would substantially improve transparency and reproducibility.

Ethics and reporting transparency
Although the data are described as de-identified, the manuscript does not include a clear ethics or IRB statement specifying approval, exemption, or waiver of consent. Given the use of human clinical samples, this information should be explicitly reported.
If a reporting summary or checklist is available, its inclusion would further improve transparency.

Overall assessment
This is a strong and timely manuscript with clear potential impact, particularly for low-resource diagnostic settings. The core results are compelling and support publication after minor revision, primarily focused on improving methodological clarity, transparency, and contextualization rather than adding new experimental data.

(Remarks on code availability)
Partially. I inspected the repository contents and README, but I did not execute/install the code end-to-end (the repository does not provide a complete runnable environment, dependencies list, pretrained weights, or example datasets sufficient to reproduce the full pipeline out of the box).

Assessment of the code (reproducibility and community usability)

What is available / positives

The repository is publicly accessible and contains Python code and Jupyter notebooks associated with the ALLocate analysis, including:

a region_cnn/ directory (ROI CNN training assets) and

notebooks for plotting/visualizing performance, plus a central script (ALLocate_software.py).
GitHub

This is a positive step toward transparency, particularly for readers who want to understand or re-create portions of the ML evaluation workflow.

Documentation quality / usability

The README is very brief and currently provides only a high-level statement about where region-CNN training files and plotting/visualization code live. It does not include:

installation instructions (e.g., environment setup/conda/pip),

a requirements.txt/environment specification,

step-by-step commands to run training/inference,

how to obtain/format the datasets,

pretrained weights or model checkpoints,

expected input/output directory structure.
GitHub

There are no releases/packages and no clear “quick start” pathway for new users.
GitHub

Extent to which results are reproducible

Partial reproducibility is feasible for readers already familiar with Python/ML workflows who can infer dependencies and who have access to similar data.

However, full reproducibility of the paper’s end-to-end results is currently limited because the repository does not appear to provide:

the curated annotated datasets used in the paper (or a public substitute with identical preprocessing),

a complete, documented pipeline tying together acquisition → tiling → ROI selection → YOLO inference → slide-level aggregation,

the hardware acquisition/control code (stage/camera integration) and associated configuration details.

Overall judgment

The repository is a useful supplemental resource (especially for understanding some parts of the analysis and plotting), but it is not yet a fully usable community resource for reproducing the complete study without substantial additional documentation and artifacts.

Suggestions to improve code value and reproducibility (for authors)

Add a requirements.txt / environment.yml, plus OS/GPU/CPU notes.

Provide a Quick Start section with exact run commands.

Provide pretrained weights (or instructions to download them) and example config files.

Include a small toy/sample dataset and expected file structure (or scripted download + preprocessing).

Provide an explicit “reproduce paper figures/tables” guide.

If possible, add documentation for the acquisition/control stack (camera control, stage scan logic, synchronization).

Version 1:

Reviewer comments:

Reviewer #1

(Remarks to the Author)
Thank the authors for the very detailed response. All my concerns have been addressed and I have no further comments.

(Remarks on code availability)
Package (tqdm) is missing from the requirements. Data files are not present for reproducibility (running notebooks).

Reviewer #2

(Remarks to the Author)
Dear Authors,

Thank you for your comprehensive and carefully detailed reply to the comments. The revisions you have carried out address the main concerns raised during the initial evaluation. In particular, the enlargement of the dataset, the clearer presentation of the methodology, and the strengthening of the external validation cohort greatly enhance the overall robustness and clarity of the study.
I would also like to emphasize this low-cost microscopic system, based on robust methods with minimal computational requirements, represents a meaningful strength of the study and may offer valuable perspectives for implementation in rapid screening settings, especially in regions or institutions with limited resources.
As you appropriately indicate in the revised manuscript, such an approach still requires expert review to obtain a valuable diagnosis. In this context, I would suggest slightly softening the statement in the abstract. For example: “These results suggest that ALLocate provides an accurate, generalizable, and cost-effective approach bridging the gap between AI innovation and practical deployment for marrow smears screening in underserved regions »

Sincerely,

(Remarks on code availability)

Reviewer #4

(Remarks to the Author)
I am invited as a replacement reviewer for previous reviewer 3, who was not responsive during the revision. I read through previous reviewer 3 's comments and read through the responses from the authors. I find that all comments have been properly addressed, except one minor question: the overall specifiication is much better described, but to really support reproducibility, should the customized 3D print files also be released and made available as part of the publication?

(Remarks on code availability)

Open Access This Peer Review File is licensed under a Creative Commons Attribution 4.0 International License, which permits use, sharing, adaptation, distribution and reproduction in any medium or format, as long as you give appropriate credit to the original author(s) and the source, provide a link to the Creative Commons license, and indicate if changes were made.
In cases where reviewers are anonymous, credit should be given to 'Anonymous Referee' and the source.
The images or other third party material in this Peer Review File are included in the article’s Creative Commons license, unless indicated otherwise in a credit line to the material. If material is not included in the article’s Creative Commons license and your intended use is not permitted by statutory regulation or exceeds the permitted use, you will need to obtain permission directly from the copyright holder.
To view a copy of this license, visit https://creativecommons.org/licenses/by/4.0/

Reviewer's Report (Consolidated and Revised)

Summary

This manuscript presents **ALLocate**, a low-cost, AI-enabled self-driving microscope system for automated detection of acute myeloid leukemia (AML) from bone marrow smear slides. The system integrates a 3D-printed, motorized XY stage with a two-stage machine-learning pipeline consisting of region-of-interest (ROI) classification and YOLOv8-based blast detection. The authors validate the approach across internal datasets, independent external cohorts, and—importantly—on physical glass slides. The work addresses a clinically relevant and globally important problem and demonstrates a compelling end-to-end proof of concept.

Overall, the manuscript is technically sound and presents strong experimental results. However, several aspects related to system architecture clarity, reproducibility of the hardware implementation, ethics reporting, and positioning within the broader ecosystem of edge-based microscopy require clarification or expansion.

Major Strengths

1. **End-to-end validation including physical slides**
   The demonstration of the complete hardware–AI pipeline on real glass slides significantly strengthens the translational relevance of the work and distinguishes it from purely software-based studies.
2. **Cost-aware design with deployment relevance**
   The reported hardware cost (~USD 150, excluding the microscope) is compelling and addresses a major barrier associated with whole-slide imaging systems.
3. **External validation across institutions**
   Training on UCSF data and testing on independent MSKCC cohorts improves confidence in generalizability.
4. **Clinically meaningful evaluation framework**
   Slide-level diagnosis using established blast percentage thresholds (≤5% for benign, ≥20% for AML) appropriately mirrors clinical practice.

Major Comments

1. System architecture and camera specification are insufficiently described

While the manuscript clearly describes the motion-control electronics (Arduino Mega, RAMPS, stepper motors), the overall system architecture is not explicitly stated. In particular:

- The camera model and specifications (sensor type, resolution, interface) are not reported.
- It is unclear whether image acquisition and AI inference are performed on a PC/laptop or an embedded computing platform, and how this component interfaces with the microcontroller-based motion system.
- The division of responsibilities between the microcontroller (motion control), computing hardware (image capture and inference), and software stack should be explicitly described.

This information is important for reproducibility and for assessing real-world deployability.

2. Stage actuation mechanism lacks sufficient mechanistic detail

The description of the automated stage provides a list of components but does not adequately explain how stage actuation is implemented in practice. Missing details include:

- How the stepper motors mechanically couple to the microscope stage knobs (e.g., direct coupling, gearing, belt-driven) – consider incorporating a more illustrative diagram in Figure 1 A.
- Motion resolution (e.g., effective linear displacement per motor step, microstepping configuration)
- Backlash, repeatability, or positioning accuracy considerations
- Scan pattern (e.g., raster or serpentine) and step size relative to the camera field of view
- Synchronization between stage motion and image acquisition

As written, the description is sufficient to understand *what components are used*, but not *how the stage actuation operates*. A brief schematic, flowchart, or expanded textual description would substantially improve reproducibility.

3. Firmware and tile acquisition logic are only described at a high level

The manuscript states that an “Arduino C++ script” is used to move the stage, but provides no information on:

- Firmware logic or control flow
- Timing and dwell behavior at tile locations
- How scans are initiated, terminated, or parameterized

Similarly, tile acquisition is described conceptually but not algorithmically. Clarifying whether scanning follows a fixed raster pattern, how tile overlap is handled, and how acquisition relates to downstream analysis would improve transparency.

4. Z-axis focusing and focus maintenance are not implemented or described

No description is provided for Z-axis actuation or autofocus, nor is the frequency of refocusing during tile acquisition specified. The system appears to operate with a fixed focus, which the authors acknowledge as a limitation in the Discussion. This limitation should be stated explicitly in the Methods to avoid ambiguity and to clarify performance expectations for uneven or thick smears.

5. Ethics approval and human-subjects oversight are not explicitly stated

Although the datasets are described as de-identified, the manuscript does not include a clear ethics or IRB statement specifying:

- Whether IRB approval or exemption was obtained
- Which institutions approved the data use
- Whether informed consent was waived

Given the use of human clinical samples and cross-institutional data, a formal ethics statement is required.

6. Limited discussion of edge-based and open-source microscopy literature

The Discussion appropriately contextualizes the AI performance relative to prior bone marrow analysis methods, but it does not sufficiently engage with existing literature on low-cost, edge-based, or open-source digital microscopy systems. Notably:

- ALLocate is not explicitly framed within the broader trend toward edge AI or decentralized microscopy.
- No references are provided to established open-source microscope platforms; only a single stage adapter design is cited.

Given that hardware–AI integration and affordability are central contributions, broader contextualization would strengthen the manuscript.

7. Scope of disease and workflow completeness

The system is evaluated specifically for AML versus benign cases, yet the framing occasionally refers more broadly to “acute leukemia.” This distinction should be clarified.

In addition, while the system is positioned as an assistive tool, the manuscript does not describe an implemented mechanism for human expert review of ML outputs (e.g., reviewing YOLO-labeled cells or correcting errors). Incorporating this as a future improvement in the Discussion would strengthen the clinical positioning of the system.

Minor Comments

- Camera exposure control, illumination stability, and imaging consistency are not discussed.
- Failure cases (e.g., misclassification of immature granulocytes) are mentioned but not illustrated.
- Some supplementary tables are dense; highlighting final model configurations would improve readability.

Recommendation

Minor Revision

The manuscript presents a strong and timely contribution with clear translational relevance. Addressing the points above—particularly those related to hardware actuation clarity, system architecture, ethics reporting, and broader contextualization—would significantly improve reproducibility, interpretability, and impact without requiring additional experiments.

Overview:

We thank the editor and reviewers for their thoughtful and constructive comments. We have carefully addressed all points raised and revised the manuscript accordingly.

Below, we provide a detailed point-by-point response, organized by Reviewer 1, Reviewer 2, and Reviewer 3. Each comment has been addressed individually. Reviewer comments are presented in **Arial**, our responses in **Times New Roman**, and corresponding revisions from the manuscript are ***italicized***. A revised version of the manuscript with track changes is also provided.

In response to the reviewers' suggestions, we have made substantial revisions throughout the manuscript. The figures and tables have been updated accordingly; the revised manuscript now includes 5 main-text figures, 1 table, 9 supplementary figures, and 14 supplementary tables.

For Reviewer 1, we have improved clarity in model evaluation and reporting by expanding methodological descriptions, refining evaluation protocols, and adding additional quantitative analyses to improve interpretability and reproducibility.

For Reviewer 2, we have addressed concerns regarding dataset size, heterogeneity, and external validation by substantially expanding both the training dataset and evaluation cohorts. This includes a significant increase in region-level and cell-level annotations, retraining all models, and incorporating an independent external dataset from MSKCC, increasing the number of physical glass slides evaluated from 36 to 165 across two institutions. We have also clarified the diagnostic scope of ALLocate and expanded the Discussion to outline its limitations and intended clinical use.

For Reviewer 3, we have expanded the Methods and Discussion sections to provide detailed descriptions of the hardware system, acquisition workflow, and relevant literature context. We have also improved the reproducibility and usability of the code repository by adding documentation, dependency specifications, and example workflows.

Reviewer #1:

In this study, Yan and Sun et al developed ALLocate, an affordable, AI-powered microscope workflow that enables accurate and efficient leukemia detection. The effective design of a CNN-based region classifier followed by a YOLOv8-based cell detection and classification frees up the pipeline from the need of whole-slide images. The authors designed comprehensive evaluation tasks and demonstrated impressive model performance at every stage of the pipeline. Overall, it is an innovative tool with great potentials for leukemia detection in a resource-limited real-world setting.

My minor comments fall into the following 3 categories:
Comment 1: The results are convincing, but some technical details in model evaluations are missing. Adding those could further strengthen the paper, help readers who are not familiar with some assumptions in the leukemia world, and make sure the work can be reproduced, e.g.

Response: Thank you for your thoughtful review and constructive comments. We provided a detailed point-by-point response to ensure that all concerns are carefully addressed and that the revised manuscript is clearer, more rigorous, and fully reproducible.

Comment 2: Assuming the CNN region classifier should be a multiclass model, rather than a multilabel model. It would be nice to explicitly mention that. Therefore it will be clear that the precision recall was computed using the predicted class (argmax) rather than a pre-defined binarization threshold. It would be helpful to provide the prevalence of the 3 classes, and confusion matrix to help understand the model behavior.

Response: Thank you for this helpful clarification. The CNN region classifier is indeed formulated as a multiclass classification problem rather than a multilabel task. The network was trained using a softmax output layer with cross-entropy loss, which enforces a probability distribution across the three classes (summing to 1). During inference, the predicted class label is obtained using the argmax of the softmax probabilities, and precision–recall metrics were computed based on these discrete class predictions rather than thresholded binary outputs.

We agree that reporting class prevalence and the confusion matrix would improve interpretability of the model behavior. In the revised manuscript, we have:

a. Explicitly state the multiclass formulation and training setup. See our changes at lines 384–389.

"The CNN region classifier is formulated as a multiclass classification problem. The network was trained using a softmax output layer with cross-entropy loss, which enforces a probability distribution across the three classes (summing to 1). During inference, the predicted class label is obtained using the argmax of the softmax probabilities, and precision–recall metrics were computed based on these discrete class predictions rather than thresholded binary outputs."

b. Report the prevalence (sample counts and percentages) of the three classes in the evaluation dataset in **Supplementary Table S14**, see below.

**Class**	**Train**	**Test**	**Total**	**Prevalence (%)**
Adequate	3,386	846	4,232	37.01%
Blood	3,386	846	4,232	37.01%
Clot	2,377	594	2,971	25.98%
**Total**	9,149	2,286	11,435	100%

Table S14. Class distribution and prevalence in training and test datasets

c. Add a confusion matrix to illustrate per-class performance in **Supplementary Figure S9**, see below.

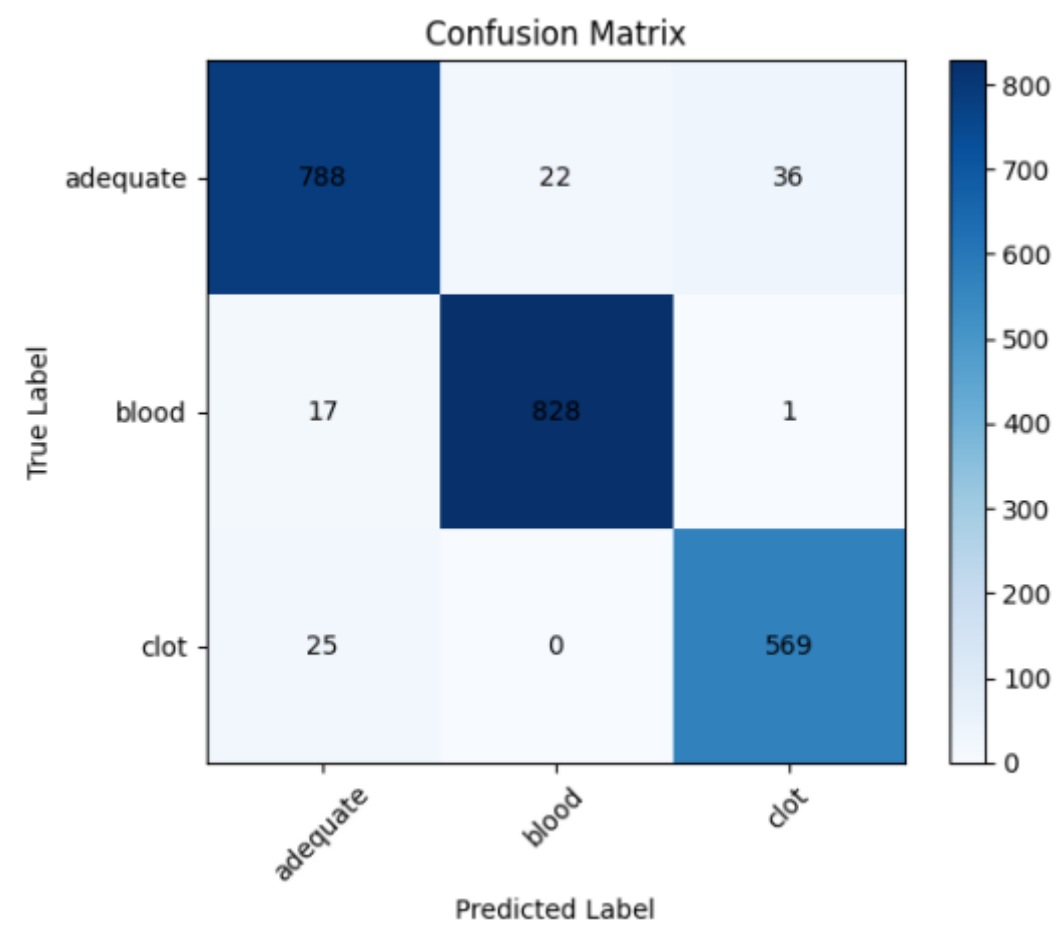

Figure S9. Confusion matrix of region classifier performance

Comment 3: For YOLOv8 performance evaluation, figure 3B, how were the precision and recall determined? Using a balanced metric? Can you include some discuss around how the trade-off in real-world application factor into the algorithm design here?

Response: We thank the reviewer for this comment. We have clarified this information.

Here is the clarification; it can be found at lines 418–421.

"Precision and recall for the YOLOv8 model were computed on the validation set using standard object-detection evaluation protocols at a predefined confidence threshold (confidence = 0.25), selected based on optimal performance on the validation cohort. Once an object was detected, class assignment was determined by the argmax of class probabilities (blast vs. non-blast)."

Balanced composite metrics were not used because performance was evaluated at the cell level and reported separately for each class. This approach is consistent with object-detection practice and avoids obscuring clinically relevant behavior (e.g., sensitivity to blasts) that could be masked by aggregated or class-balanced metrics. We have added clarification at lines 422–425.

We have included a discussion on real-world deployment considerations, associated trade-offs, and how the model is used in practical settings.

Real-world deployment and trade-offs. This can be found at lines 300–305.

"Regarding real-world deployment considerations, the algorithm design intentionally reflects a trade-off between sensitivity, precision, and operational speed. The system is intended as a rapid triage and screening tool integrated with an automated microscope, where missed blasts would be clinically more consequential than a moderate increase in false positives. Therefore, the detection threshold was tuned to maintain high sensitivity for blast identification while preserving acceptable precision to avoid excessive downstream review burden."

Practical use of the model. This can be found at lines 305–309.

"In practical settings, ALLocate operates as an assistive system rather than a standalone diagnostic replacement: detected regions and cell counts are reviewed in aggregate, and outputs can be verified by a clinician. This design prioritizes rapid identification of potentially malignant regions, efficient slide prescreening, and reduction of manual workload while maintaining clinically meaningful accuracy."

Comment 4: - It is unclear how the pathologist performance (auroc) was derived from Figure 3E. Is there a reference pathologist for 'ground truth' annotation, and an evaluation pathologist? Is number of blast used here for classification?

Response: We thank the reviewer for identifying this point of confusion. We agree that the original figure caption did not clearly describe how AUROC was computed for both ALLocate and the pathologist, and we have revised the manuscript accordingly.

For this analysis, region-level ground-truth labels (AML-associated vs. benign) were established by expert hematopathologists (lines 439–443).

"A region was labeled as AML-associated only when the reviewing hematopathologist determined it to contain leukemic features and the assessment was consistent with the corresponding slide-level clinical diagnosis. Regions without leukemic features and concordant with benign slide-level diagnosis were labeled as benign. These curated region-level annotations served as the reference standard for AUROC evaluation."

To generate ROC curves, both the model and the pathologist produced quantitative region-level blast enrichment scores:

- Is number of blast used here for classification?

For each region, YOLOv8 detected nucleated cells and classified them as blast or non-blast. The blast ratio (number of blasts divided by total detected nucleated cells) was used as a continuous prediction score.

- how the pathologist performance (auroc) was derived from Figure 3E?

A board-certified hematopathologist independently annotated the same regions, labeling normal and blast cells. From these annotations, a blast ratio was similarly computed for each region. These continuous blast ratios (from BOTH the model and the pathologist) were then compared against the binary ground-truth label (AML vs. benign region) to derive AUROC.

Both evaluated against the same ground-truth labels.

We have revised:

- The Figure 3E caption to explicitly describe this evaluation framework.

  "E. ROC curves comparing ALLocate and pathologist performance for region-level AML classification on the external validation cohort (EVC). Ground-truth labels were curated by expert hematopathologists. Prediction scores were defined as blast ratios (blast cells / total nucleated cells) for both the model and an independent pathologist."

- The Results section to clarify that blast ratio was used as the continuous classification score at lines 147–152.

  "At the region level, ALLocate achieved an AUROC of 0.9012 for distinguishing benign regions from AML-associated regions, compared to 0.9359 for the hematopathologist, using blast ratio as the continuous prediction score (Fig. 3E). Quantitative analysis of blast counts showed that, in normal regions, ALLocate detected a similar number of blasts as the pathologist (mean 0.75 vs. 0.60 blasts per tile; Fig. 3F, G) without significant statistical difference (P=0.07)."

Comment 5: The authors need to pay more attention to the figure legends and ensure the panel descriptions match the figures, and the information in the figure is self-sufficient, e.g.

- Figure 1A, it would be helpful to add (1)-(5) in the figure to help reader understand the hardware setup. The ALLocate price in the panel ($100) does not align with the main text ($150)
- Figure 5, the figure legend for the panels do not match the content - one extra panel and the order is incorrect
- More effective visualizations could be used to highlight the message, e.g.
- Figure 3B bar plot with error bars - side note: confidence intervals should be consistently reported especially given the very high model performance
- Figure 3G - it is unclear whether paired samples are used in the analysis, then paired visualization should be used, e.g. swarmplot with connected dots or scatter plot

Response: We thank the reviewer for these detailed and constructive suggestions. We have revised all the suggestions point to point. We have also carefully reviewed all figures and legends throughout the manuscript to ensure consistency, accuracy, and that no further discrepancies remain.

Figure 1A: We have added numbered labels (1–5) to the hardware diagram to clearly indicate the main components and workflow of the ALLocate setup. We also corrected the reported cost. The mechanical system cost is an estimate that typically ranges between $100–$150 depending on component sourcing; this is now consistently reported as ~$150 across the figure and main text for clarity.
Figure 5: We apologize for the mismatch between panel ordering and the legend. The figure legend has been rewritten and reordered to accurately correspond to each panel, and the extra/misaligned panel reference has been removed.
Figure 3B: We have added error bars and now report confidence intervals for the performance metrics.

"B. YOLOv8 performance for detecting non-blast cells and blasts, including precision, recall, mAP50, and precision–recall curves. Error bars represent ±1 standard deviation across cross-validation folds; corresponding 95% confidence intervals are: precision [0.864–0.870], recall [0.849–0.867], and mAP50 [0.926–0.928] for non-blast cells, and precision [0.805–0.821], recall [0.816–0.844], and mAP50 [0.856–0.870] for blasts."

Figure 3G: To better reflect this structure, we have updated the visualization to a paired representation (swarm/scatter plot with connected points).

Reviewer #1 (Remarks on code availability):

The code is pretty readable and seems to contain the bulk of the results. However, dependencies are needed to set up the repo and run the code.

> **<u>Response:</u>** We thank the reviewer for this comment. We have improved the reproducibility and usability of the code repository by adding documentation, dependency specifications, and example workflows.

Reviewer #2 :

This article proposes an approach aimed at democratizing AI-based automatic leukemia detection on whole bone marrow smears through a low-cost, AI-powered automatic microscope scanner called ALLocate. The AI module combines a CNN region classifer with a YOLOv8-based leukemia detector. Training and validation were conducted on a private monocentric dataset of 55 digitalized whole marrow smears, while testing was perfomed on 40 external digitalized slides. Finally, 36 monocentric marrow smear slides were used for combined hardware+ AI component evaluation (i.e. End-to end evaluation). ALLocate achieved an AUC >0.99 for region-of-interest detection, a mean average precision (mAP50) >0.9 for cell detection and an overall diagnostic accuracy of 89% at the glass slide level.

AI-based algorithms for cancer detection and classification, including leukemia detection on blood/marrow smears, have already been published with remarkable performance. However, major challenges such as limited generalizability and poor explainability remain major obstacles to their clinical adoption. In my opinion, this work does not adequately address these issues and remains essentially another proof of concept study with significant limitations (particularly the very small size of the datasets) and limited novelty, apart from the implementation of a low-cost microscope scanning system.

Response: Thank you for your thoughtful and constructive feedback. We appreciate your comments and believe they have significantly strengthened the manuscript. In response, we have undertaken substantial revisions to address the key concerns raised. Specifically, we have expanded the dataset at both the region and cell levels, clarified the number of cases and patient-level distributions, and performed a substantially expanded external validation using an independent MSKCC cohort (n = 129; 73 normal, 56 AML), alongside the original UCSF cohort (n = 36; 18 normal, 18 AML), yielding a total of 165 cases. In addition, we have improved the characterization of AML subtype heterogeneity, clarified methodological details including data partitioning strategies, and strengthened the evaluation of the end-to-end hardware–AI system in a fully external setting.

These revisions substantially improve the scale, transparency, and generalizability of the study, and we hope the following point-by-point responses address your concerns.

Major comments :

Comment 1: Limited dataset size and heterogeneity:

The number of cases used for training, validation, and testing is unclear and appears surprisingly limited for a CNN-based machine learning approach.

> **<u>Response:</u>** We thank the reviewer for raising this important concern regarding dataset size and heterogeneity. In response, we have made substantial efforts to expand both the training and evaluation datasets.

Dataset Purpose		Inst.	Hardware	Data Type	Tasks	Qty	Patients
TR	Training & Evaluation	UCSF	40x/400x-equivalent WSI images (Aperio AT2, GT450)	Region Tiles	Model development	9,149	70
ER		UCSF	40x/400x-equivalent WSI images (Aperio AT2, GT450)		Adequacy evaluation	2,286	
TC	Training & Evaluation	UCSF	40x/400x-equivalent WSI images (Aperio AT2, GT450)		Model development	109,825	120
EC		UCSF	40x/400x-equivalent WSI images (Aperio AT2, GT450)		Cell classification	26,533	
EVC	External Validation	MSKCC	40x/400x-equivalent WSI images (Hamamatsu S360, S20)		Malignancy evaluation	186	95
EVS		MSKCC	40x/400x-equivalent WSI images (Hamamatsu S360, S20)	Digital Slides	Malignancy evaluation	40	40
SDM	Self Driving Microscope	UCSF	40x/400x self-driving compound microscope (Olympus BX40, 3D printed actuators, Arduino controller)	Glass Slides	Malignancy evaluation	36	36
		MSKCC	40x/400x self-driving compound microscope (Olympus BX46, 3D printed actuators, Arduino controller)		Malignancy evaluation	129	129

Specifically, the region classification dataset has been expanded from approximately 1,000 to over 10,000 annotated regions (Table 1, Supplementary Table S1). In addition, the cell-level annotation dataset has been significantly increased from approximately 25,000 to over 140,000 annotated cells (Table 1, Supplementary Table S1, Table S12).

We have also clarified the number of slides used across all stages of the study. The revised dataset now includes:

Table S12. Breakdown of case-level datasets used in this study

Task/Dataset Description	Institution	Number of cases
Region classification	UCSF	70
Cell detection	UCSF	190
Region + cell detection (external evaluation)	MSKCC	95
Digital slides (external evaluation)	MSKCC	40
Physical slides (internal evaluation)	UCSF	36
Physical slides (external evaluation)	MSKCC	129

In total, this corresponds to 560 slides, which are now explicitly reported in Supplementary Table 12 and described in the **Methods** section (lines 350–380).

"*The dataset used for training, validation, and evaluation of the deep learning models was acquired from de-identified bone marrow smear whole-slide images provided by the Department of Laboratory Medicine at the University of California, San Francisco (UCSF)*

[23] (Fig. 1C). All slides were scanned at 40× magnification using Aperio AT2 and GT450 scanners without additional Z-axis focusing.

For region classification, a total of 9,149 region tiles were used for training (TR), with an additional 2,286 tiles reserved for evaluation (ER), ***derived from 70 patient slides (n_slides = n_patients)****. For cell detection, 109,825 annotated cell instances were used for training (TC), and 26,533 instances were used for evaluation (EC),* ***derived from 190 patient slides (n_slides = n_patients)****. Model development (training and validation) for both tasks was performed at the region level, as annotations were generated at the tile level and multiple regions were extracted from each slide.*

...

External validation was performed using de-identified datasets from Memorial Sloan Kettering Cancer Center (MSKCC), scanned at 40× equivalent magnification using Hamamatsu S360 and S20 scanners. This included 186 region tiles (99 normal, 87 AML) for partial pipeline evaluation (EVC), ***derived from 95 patient slides (49 AML, 46 normal; n_slides = n_patients)****, and 40 digital slides (20 normal, 20 AML) for full pipeline evaluation (EVS).*

To evaluate the end-to-end performance of the integrated hardware–AI system, physical glass slides were tested using the self-driving microscope platform. Internal evaluation at UCSF included 36 slides (18 normal, 18 AML), while external evaluation at MSKCC included 129 slides (73 normal, 56 AML). ***All slide-level evaluations were performed at the patient level, with each slide corresponding to a unique patient (i.e., n_slides = n_patients), and all external validation cohorts were fully independent and not used during model training.*** *These experiments assessed system performance under realistic clinical workflow conditions, including automated scanning and blast quantification."*

We would also like to note that, unlike many CNN-based studies that rely on weakly supervised learning with slide-level labels or coarse region-level annotations, our dataset includes dense annotations at both the cell and region levels, enabling more granular and biologically meaningful modeling.

We appreciate the reviewer's feedback, which has helped us significantly strengthen the dataset and improve clarity in reporting.

Comment 2: AML is a heterogeneous group with multiple differentiation and molecular subtypes leading to significant morphological variability. This heterogeneity is not discussed in this paper (which AML subtypes were used?) and cannot be reflected in these limited datasets.

Response: Thank you for your feedback. We agree that AML encompasses a wide range of molecular and morphological subtypes. A detailed breakdown of subtype distribution (with case counts) has been added to **Supplementary Table 13** and described at lines 361–366, demonstrating that the dataset captures a broad spectrum of AML heterogeneity.

"The AML cohort included a diverse range of molecular and morphological subtypes encountered in routine clinical practice, including AML with RUNX1::RUNX1T1 fusion, AML with CBFB::MYH11 fusion, AML with KMT2A-rearrangement, AML with NPM1-mutation, AML with TP53-mutation, therapy-related AML, AML transformed from myeloproliferative neoplasms, and AML with myelodysplasia-related changes. A detailed subtype distribution is provided in Supplementary Table 13."

**Type**	**Prevalence**
AML with RUNX1::RUNX1T1 fusion	6
AML with CBFB::MYH11 fusion	2
AML with KMT2A rearrangement	6
AML with MECOM rearrangement	3
AML with NUP98 rearrangement	1
AML with NPM1 mutation	7
AML with CEBPA mutation	1
AML, myelodysplasia-related	12
AML with TP53 mutation	5
Therapy-related AML	5
AML transformed from MPN	3
AML with germline DDX41 mutation	1
AML with FLT3	5
AML with IDH2	1
AML, NOS	12

We acknowledge that not all cases have complete molecular annotations, and we have added this as a limitation in the Discussion (lines 271–274).

"It is important to note, however, that our dataset captures a range of AML subtypes, the sample size within each subtype remains limited, and future studies with larger, subtype-balanced cohorts are needed to fully evaluate model performance across AML variants."

Comment 3: Limited diagnostic scope and lack of prediction confidence:
The dataset includes only AML and normal marrow smears. This binary classification task is overly simplistic and does not reflect real diagnostic practice, where cytologists and pathologists must differentiate among a broad spectrum of diseases (e.g., acute lymphoblastic leukemia, myelodysplasia, chronic MPNs, lymphomas, metastases). What prediction would ALLocate generate on these out of distribution input data ? (meaning non-AML and non-normal samples).
Moreover, the system provides no confidence index for its predictions, making it difficult to assess their reliability.

Response: We agree that real-world hematopathology involves a wide spectrum of diseases beyond AML and normal marrow. Our current work focuses specifically on AML blast detection and screening, which represents a narrower but clinically important task.

The primary goal of ALLocate is not to provide a full hematologic differential diagnosis, but rather to serve as a rapid screening and triage tool, **particularly in resource-limited environments where expert hematopathologists may not be immediately available.** Acute myeloid leukemia is one of the most urgent hematologic malignancies, requiring rapid recognition and immediate treatment initiation, where delays can have significant clinical consequences. Therefore, a system capable of rapidly identifying blast-enriched smears suggestive of acute leukemia can have meaningful clinical value even without providing a full diagnostic classification.

For samples containing other hematologic diseases (e.g., myelodysplastic syndromes, lymphoid malignancies, or metastatic involvement), ALLocate would still produce blast detection outputs and blast proportion estimates, as these are directly derived from cell-level detection. While these outputs alone are insufficient to establish a definitive diagnosis, they can still provide useful quantitative blast counts that may assist clinicians in identifying abnormal specimens that require expert review.

We acknowledge that evaluating performance on out-of-distribution disease categories would be an important next step. We have added a discussion of this limitation in the revised manuscript and highlight future work aimed at expanding the training dataset to include additional hematologic conditions and performing broader multi-class classification (lines 310–317).

"In addition, the current study focuses on distinguishing AML from normal marrow and does not explicitly evaluate performance on other hematologic or metastatic disease categories. For out-of-distribution samples, ALLocate would still generate blast detection outputs and quantitative blast proportion estimates; however, these outputs alone are insufficient for definitive diagnosis and should be interpreted as screening indicators requiring expert review. Furthermore, prediction confidence is inherently provided within the pipeline: the cell-level detector outputs class probabilities, while the blast proportion serves as a continuous, slide-level score that can be used for threshold-based decision-making."

Since our study does not involve survival analysis, we interpret the reviewer's request for a "confidence index" as referring to prediction confidence associated with the model outputs.

In the ALLocate pipeline, prediction confidence is provided at multiple levels. At the cell level, the YOLOv8 detector assigns a confidence score to each detected object, representing the model's estimated probability for the predicted class (blast vs. non-blast). At the region and slide levels, predictions are derived from the blast proportion (number of detected blasts divided by total nucleated cells), which serves as a continuous score used to compute ROC curves and classification thresholds (Figure 3E, Figure 4D, Figure 5G).

Comment 4: Need to clarify the methodology :
The number of cases used is unclear. It would be better to specify the number of WSIs and the number of patients rather than referring to "regions". Particularly, it would be relevant to precise the number of WSI and patients included in the different datasets in Table 1.
For ROI and cell detection tasks, the methods does not specify whether the train/test split was performed at the patch, slide, or patient level (the latter being the most appropriate to avoid data leakage).

Response: We thank the reviewer for this important comment regarding methodological clarity and data partitioning.

First, we have revised the manuscript to explicitly report the number of whole-slide images (WSIs) and patients included in each dataset. In our study, each WSI corresponds to a unique patient (i.e., n_WSI = n_patients), as each slide was derived from a distinct individual. These details are now clearly provided in **Table 1**, **Supplementary Table 12**, and the Methods section (see response to **Reviewer 2, Comment 1** for the revised Table 1), along with instance-level counts.

Regarding the data splitting strategy, we clarify that model development (training and validation) for the region classification and cell detection tasks was performed at the region level, as annotations were generated at the tile level and multiple regions were extracted from

each slide. This approach allowed efficient utilization of the densely annotated data for model optimization.

Importantly, to mitigate concerns regarding potential data leakage, we emphasize that all key performance evaluations were conducted on independent datasets. The UCSF physical slide evaluation was performed at the patient level, with each glass slide originating from a distinct patient. Furthermore, all external validation experiments were conducted on completely independent cohorts from MSKCC (**Figure 4, Figure 5**), collected at a separate institution using different scanners and clinical workflows, and not used at any stage of model training. These evaluations span region-level, digital slide-level, and physical slide-level analyses, including the end-to-end hardware validation added in this revision.

We believe that this multi-level, multi-institutional external validation provides strong evidence of model generalizability and substantially mitigates concerns related to internal data partitioning. We acknowledge that patient-level partitioning within internal datasets represents a more stringent evaluation strategy and will be incorporated in future studies with expanded cohorts.

We have revised the manuscript to clearly describe the data splitting strategy (lines 355–361):

"For region classification, a total of 9,149 region tiles were used for training (TR), with an additional 2,286 tiles reserved for evaluation (ER), derived from 70 patient slides (n_slides = n_patients). For cell detection, 109,825 annotated cell instances were used for training (TC), and 26,533 instances were used for evaluation (EC), derived from 190 patient slides (n_slides = n_patients) (Supplementary Table 12). Model development (training and validation) for both tasks was performed at the region level, as annotations were generated at the tile level and multiple regions were extracted from each slide."

Comment 5: Lack of external validation for the self driving microscope task :
The final end-to-end evaluation of ALLocate was performed only on a limited number (n=36) of internal, monocentric slides. Validation on external cohorts, preferably from multiple centers, is essential to demonstrate the robustness of ALLocate. In fact, interlaboratory variability can significantly affect the generalizability of such AI systems, especially when trained on a monocentric dataset (which is the case here).

Response: We thank the reviewer for this important comment regarding external validation of the end-to-end system.

In response to the reviewer's suggestion, we have substantially expanded the external validation of the full hardware–AI pipeline. Specifically, the ALLocate system was deployed at Memorial Sloan Kettering Cancer Center (MSKCC), where we evaluated the system on 129 independent physical marrow smear slides (73 normal, 56 AML) acquired using the self-driving microscope. These slides were obtained from a separate institution and patient cohort and were not used at any stage of model training.

This expansion represents a more than threefold increase in the number of physical slides and, importantly, converts the end-to-end evaluation into a fully external, multi-institutional validation setting.

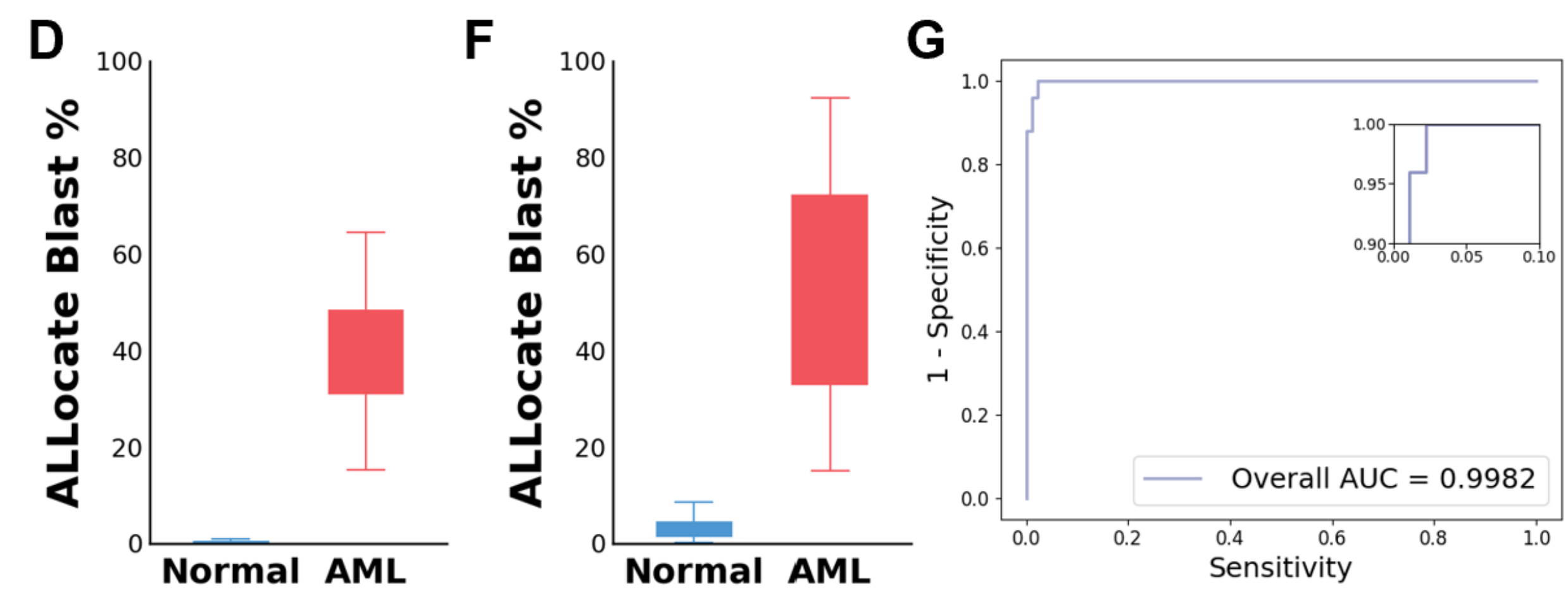

D. Overall statistics for blast percentage detection by ALLocate on the UCSF SDM dataset. F. Overall statistics for blast percentage detection by ALLocate on the external MSKCC SDM dataset. G. ROC curve for the combined UCSF and MSK SDM dataset detections by ALLocate. Ground-truth labels correspond to slide-level diagnoses, and prediction scores are defined by the estimated blast counts produced by ALLocate.

"To comprehensively evaluate the entire ALLocate pipeline, including both hardware components and AI modules, we processed a total of 165 glass bone marrow smear slides, including 36 slides (SDM, UCSF) from UCSF and ***an additional 129 slides (SDM, MSKCC) from MSKCC through the complete system.*** *Moving from the region to the slide level, we applied the diagnostic logic of ALLocate in a manner that mimics the pathologist's evaluation process (Figure 5A). ALLocate was installed on a microscope and applied to physical slides, with its AI component generating blast percentages. Each clinical sample was first digitally captured by the ALLocate hardware, and the captured images were subsequently filtered by a region classifier to produce representative images with at least 500 cells for analysis.*

…

ALLocate demonstrated strong and consistent performance across both internal and external cohorts. Benign slides exhibited low blast percentages, as confirmed by both AI-based and

clinician diagnoses (Figure 5D, F). The difference in blast percentage between AML and normal samples was highly significant in both the UCSF and MSKCC cohorts ($p = 4.72 \times 10^{-10}$ and $p = 1.77 \times 10^{-23}$, respectively). In addition to statistical analysis, we applied clinically established thresholds (≤5% blasts for benign and ≥20% blasts for AML). On the internal UCSF cohort, ALLocate correctly classified 17 out of 18 AML cases (94.4%) and 18 out of 18 normal cases (100%), corresponding to an overall accuracy of 97%. Importantly, performance remained robust on the fully independent external MSKCC cohort, where ALLocate correctly classified 53 out of 56 AML cases (94.6%) and 58 out of 73 normal cases (79.5%), achieving an overall accuracy of 86.0%. This modest decrease in performance on the external cohort is expected due to differences in slide preparation, imaging conditions, and institutional workflows, and further underscores the model's robustness under real-world variability.

Consistent with the separation observed in Figure 4, the two categories are linearly separable, resulting in an AUC approaching 1.0 (Figure 5G)*. High performance across the internal and external SDM datasets highlights the system's strong diagnostic performance in an independent, multi-institutional setting."*

Moreover, validation of the AI module on public datasets would also help reinforce its generalizability.

Response: Regarding public datasets, to our knowledge, there are currently no publicly available bone marrow aspirate smear datasets with blast-level bounding box annotations suitable for object detection tasks. This reflects a broader limitation in the field rather than a constraint specific to our study. We have acknowledged this in the revised manuscript and highlighted the need for future efforts toward the development of standardized, publicly available annotated datasets for benchmarking (lines 290–294).

"In addition, to our knowledge, there are currently no publicly available bone marrow aspirate smear datasets with blast-level bounding box annotations suitable for object detection tasks. This reflects a broader limitation in the field rather than a constraint specific to this study and highlights the need for standardized, publicly available datasets for benchmarking and future model development."

Minor comments :

Comment 6: Figure panels and legends require correction.

Response: We thank the reviewer for this comment. All figure panels and legends have been carefully reviewed and revised.

Comment 7. Does ALLocate also work on peripheral blood smears ?

Response: The current study focuses specifically on bone marrow aspirate smears, and evaluation on peripheral blood smears is beyond the scope of this work. However, the underlying framework is adaptable, and extending the system to peripheral blood smear analysis represents an important direction for future work.

Comment 8. Specify which statistical tests were used.

Response: Statistical analyses used in this study include paired t-tests for comparison of blast counts between ALLocate and pathologist assessments, two-sample t-tests for comparison between predictions on normal samples and AML samples, receiver operating characteristic (ROC) curve analysis with area under the curve (AUC) for classification performance, and standard object detection metrics including precision, recall, F1 score, and mAP50. In addition, correlation analysis was performed to assess agreement between ALLocate and pathologist blast counts.

These details have now been explicitly clarified in the Methods section (lines 468–478).

"Statistical analyses were performed to evaluate agreement between ALLocate and pathologist assessments, as well as overall model performance. Differences in blast counts between ALLocate and pathologist evaluations were assessed using independent paired t-tests. Correlation analysis was performed to quantify agreement between automated and expert-derived blast counts.
Classification performance was evaluated using receiver operating characteristic (ROC) curve analysis, with area under the curve (AUC) reported. For cell detection tasks, standard object detection metrics were used, including precision, recall, F1 score, and mean average precision at an intersection-over-union threshold of 0.5 (mAP50). All statistical analyses were performed using standard scientific computing libraries. A p-value of <0.05 was considered statistically significant where applicable."

Reviewer #2 (Remarks on code availability):

Comment 9: The authors do not share their code or comment on its potential availability after publication.

Response: We would like to clarify that the code supporting this work is already publicly available. The GitHub repository includes detailed installation instructions (requirements.txt), as well as scripts for data preprocessing, model training, and evaluation. In addition, we provide Jupyter notebooks that reproduce the main figures presented in the manuscript, enabling full reproducibility of the reported results.

We thank the reviewer for their comments, which have helped us further strengthen the manuscript.

Reviewer #3 :

Summary and noteworthy results

This manuscript reports the development and validation of ALLocate, a low-cost, AI-enabled self-driving microscope system for automated detection of acute myeloid leukemia (AML) from bone marrow smear slides. The most noteworthy results are:

1. The successful integration of inexpensive, 3D-printed motorized hardware with a two-stage machine-learning pipeline.
2. Demonstration of robust performance across internal datasets, external institutional datasets, and—most importantly—physical glass slides, which is rarely shown in comparable studies.
3. Competitive blast detection accuracy (mAP ≈ 0.91) and slide-level diagnostic accuracy (~89–92%) without reliance on whole-slide scanners.

The end-to-end validation on real slides is a particularly strong and impactful aspect of the work.

Significance and relationship to existing literature

The work is highly relevant and significant to the fields of computational pathology, digital hematopathology, and global health diagnostics. It addresses a major limitation of much existing literature: the dependence on expensive whole-slide imaging infrastructure.

Relative to established literature, the manuscript:

• Compares favorably with prior deep-learning approaches for bone marrow analysis in terms of blast detection performance.

• Is distinct in its system-level contribution, combining low-cost hardware, AI inference, and real-world validation.

Comment 1: However, while the manuscript situates the AI performance well within prior hematopathology literature, it would benefit from a broader discussion of edge-based, low-cost, or open-source microscopy systems, which are increasingly relevant to the stated deployment goals.

Response: We thank the reviewer for this thoughtful and important suggestion. We agree that situating our work within the broader context of low-cost, edge-based, and open-source microscopy systems is highly relevant to the intended deployment of ALLocate.

In response, we have expanded the manuscript to include additional discussion of this literature in both the Introduction (lines 55–64) and the Discussion (lines 230–236), to better contextualize our approach within existing efforts in accessible diagnostic imaging.

Introduction (lines 55–64):

"Recent efforts have explored low-cost and edge-based microscopy platforms for hematologic and cytologic applications. For example, Li et al. developed an autonomous, low-cost microscopy system (~$250–$500) capable of detecting infectious agents in peripheral blood smears, including Loa loa, *Trypanosoma brucei rhodesiense*, *and malaria* [1,2]. *Similarly, Maturana et al. introduced a 3D-printed robotic add-on with smartphone-based imaging for malaria detection* [3]. *While these approaches, together with other low-cost digital microscopy frameworks, demonstrate the feasibility of inexpensive automated microscopy* [4, 5], *they are primarily focused on infectious disease detection and peripheral blood analysis, with limited evaluation for leukocyte classification in bone marrow smears."*

Discussion (lines 230–236):

"A key strength of our approach is its low cost, with a total system cost of less than $150 compared to approximately $300,000 for commercial whole-slide scanners. Together with other emerging low-cost digital microscopy frameworks [4, 5], *this work highlights the growing interest in accessible diagnostic imaging platforms. Combined with a CNN-based region classifier and YOLOv8-based cell detection, the proposed system provides an end-to-end solution for automated analysis of bone marrow smears and can be deployed in resource-limited clinical settings."*

1. Li, H., Lin, H., Shrestha, P., Bhansali, R., Yan, Y., Pannu, J., Marx, K., Ouyang, W., Fuentes Valenzuela, L., Li, E., Kothari, A., Nowak, J., Soto-Montoya, H., Jussupov, A., Voisin, M., Maran, K., Byaruhanga, O., Nankabirwa, J., Greenhouse, B., Jagannathan, P., & Prakash, M. (2025). Octopi 2.0: Open and scalable microscopy platform for AI-powered diagnostic applications. medRxiv. https://doi.org/10.1101/2025.03.21.25324364

2. Li, H., Soto-Montoya, H., Voisin, M., Fuentes Valenzuela, L., & Prakash, M. (2019). Octopi: Open configurable high-throughput imaging platform for infectious disease diagnosis in the field. bioRxiv. https://doi.org/10.1101/684423

3. Maturana, C. R., de Oliveira, A. D., Nadal, S., Zarzuela Serrat, F., Sulleiro, E., Ruiz, E., Bilalli, B., Veiga, A., Espasa, M., Abelló, A., Pumarola Suñé, T., Segú, M., López-Codina, D., Sayrol Clols, E., & Joseph-Munné, J. (2023). iMAGING: A novel automated system for malaria diagnosis by using artificial intelligence tools and a universal low-cost robotized microscope. Frontiers in Microbiology, 14, 1240936. https://doi.org/10.3389/fmicb.2023.1240936

4. Ocampo-López-Escalera, J., Ochoa-Díaz-López, H., Sánchez-Chino, X. M., Irecta-Nájera, C. A., Tobar-Alas, S. D., & Rosete-Aguilar, M. (2025). A low-cost platform for automated

cervical cytology: Addressing health and socioeconomic challenges in low-resource settings. Frontiers in Medical Technology, 7, 1531817. https://doi.org/10.3389/fmedt.2025.1531817

5. Choudhury, D., Dolezal, J. M., Dyer, E., Kochanny, S., Ramesh, S., Howard, F. M., Margalus, J. R., Schroeder, A., Schulte, J., Garassino, M. C., Kather, J. N., & Pearson, A. T. (2024). Developing a low-cost, open-source, locally manufactured workstation and computational pipeline for automated histopathology evaluation using deep learning. eBioMedicine, 107, 105276. https://doi.org/10.1016/j.ebiom.2024.105276

Comment 2: Support for conclusions and claims
Overall, the data presented support the main conclusions that ALLocate can accurately identify diagnostically relevant regions, detect blast cells, and distinguish AML from benign cases using a low-cost automated microscope. The use of external validation datasets strengthens these claims.
That said, some conclusions related to deployability and clinical readiness would be further strengthened by:
• Clearer description of system architecture (camera, computing platform).

Response: We thank the reviewer for this comment. We have clarified the system architecture and computing platform in the revised manuscript.

- Automated microscopy system (line 326–334):

"The ALLocate automated stage was integrated with an Olympus BX40 microscope and Olympus EP50 camera based on previously described design [22]. For external validation on MSKCC glass slides, images were acquired using an Olympus BX46 microscope equipped with an Olympus DP27 camera and CellSens software.

The system consists of custom 3D-printed components, including motor mounts, couplings, gear assemblies, and linear support structures. Two NEMA 14 stepper motors (1.8° per step, 0.4 A rated current) were used for actuation and controlled via a RAMPS 1.4 shield connected to an Arduino Mega 2560 microcontroller (Fig. 1A). The motors were powered by a 12 V, 10 A supply and driven using A4988 stepper motor drivers configured to match motor voltage requirements."

- Computing platform (line 461–466):

"Model training and primary validation were performed on a desktop workstation equipped with an AMD Ryzen 9 7900X CPU, NVIDIA GeForce RTX 4070 Ti SUPER GPU, and 32 GB RAM (Windows 11), utilizing GPU acceleration. External validation and evaluation of the automated microscopy system were conducted on a laptop computer with an Intel Core Ultra 185H CPU, NVIDIA RTX 4070 Laptop GPU, and 16 GB RAM (Windows 11), also using GPU acceleration."

Comment 3: Explicit clarification of current limitations, such as lack of autofocus and absence of a human-in-the-loop review interface.
These do not undermine the main claims but should be clearly framed as current limitations.

> **Response:** Thanks for the feedback. We have some limitation discussion in the manuscript but we agree that we should deliver it more explicitly. Specifically, we:
>
> **- Expanded the limitations paragraph in Discussion** with an explicit statement regarding the current lack of an autofocusing capability (lines 280–283).
>
> *"Although the ALLocate system achieved strong performance, several limitations should be noted. First, while the current hardware enables automated XY stage scanning for slide pre-screening, it does not include automated Z-axis focusing, which may result in focal variability across regions…"*
>
> **- Clarified the methodology** for human review and system output handling, and **acknowledged the absence** of a complete user interface (lines 283–286):
>
> *"Second, a dedicated graphical user interface for streamlined visualization, case management, and integrated pathologist review has not yet been implemented. Future work will focus on incorporating autofocus functionality and developing a unified interface to facilitate clinical integration…"*

Data analysis, interpretation, and conclusions
The data analysis is generally sound and appropriate for the study goals. Evaluation metrics (AUC, mAP, precision/recall, accuracy) are standard and well chosen. Interpretation of results is mostly balanced.
No major analytical flaws are evident. However:
Comment 4: The relatively small number of physical glass slides should be discussed more explicitly in terms of statistical power.

> **Response:** Thank you for raising this important point. In the original submission, external validation using the self-driving microscope was performed on 36 physical glass slides (18 AML and 18 normal), which we agree limited the statistical precision of the slide-level performance estimates.

To address this concern, we have substantially expanded the external validation cohort in the revised manuscript. The updated analysis now includes 129 slides from Memorial Sloan Kettering Cancer Center. This expanded multi-institutional cohort improves statistical robustness and better reflects inter-institutional variability in smear preparation and staining. For details, please see our response to **Reviewer 2, Comment 5**.

We also acknowledge that the overall cohort size remains relatively limited. Accordingly, we have added this as a limitation in the Discussion section (lines 286–290), where we note that larger, multi-center studies will be necessary to further improve statistical power and confirm generalizability.

"Also, although glass-level validation was performed on both the UCSF dataset (including an independent test set) and the MSKCC external validation cohort, the overall number of physical glass slides remains relatively limited. Larger, multi-center studies will be necessary to further improve statistical power and confirm generalizability across diverse clinical settings."

Comment 5: Failure modes (e.g., misclassification of immature granulocytes) are acknowledged but could be better illustrated.

These issues warrant revision and clarification, not rejection.

Response: Thank you for this valuable suggestion. We have expanded the Discussion (lines 294–299) to better characterize this limitation.

"Finally, we observed occasional misclassification of immature granulocytes as blasts, particularly in reactive marrow conditions. These errors most frequently occurred when immature granulocytes exhibited prominent nucleoli, a feature that can overlap morphologically with leukemic blasts and challenge the model's discriminative capacity. Future work will incorporate a more diverse set of reactive marrow samples to better capture the spectrum of granulocytic maturation and improve model robustness."

Methodology and technical soundness
The methodology is technically sound and meets expected standards for the field. The staged ML approach (ROI selection followed by cell detection) is appropriate and clinically motivated.

Comment 6: However, several aspects of the hardware and acquisition methodology require clearer description:

• The stage actuation mechanism is described largely as a list of components, without sufficient mechanistic detail.
• Firmware logic and tile acquisition strategy are only described at a high level.

Response: Thank you for highlighting the need for additional clarification regarding the hardware and firmware mechanisms underlying the ALLocate automated stage.

We have updated the Methods section to describe the movement and behavior of the ALLocate hardware and expanded it to include detailed mechanical and firmware descriptions of stage actuation and tile acquisition logic (lines 336–346).

"The system leverages the existing manual XY translation knobs of the microscope stage for motorized actuation. Custom 3D-printed couplings were designed to interface with the X- and Y-axis control knobs and secured using a combination of friction fitting and mechanical fasteners to ensure stable torque transmission. The X-axis motor is directly coupled to the stage control shaft, whereas the Y-axis motion is achieved through a geared coupling due to the coaxial arrangement of the stage controls. Both motors are mounted within a 3D-printed housing that translates with the stage and is supported by steel guide rods to maintain alignment.

Automated image acquisition is performed using a unidirectional raster scanning pattern. The stage translates along the X-axis to capture sequential image tiles, followed by an incremental Y-axis shift before scanning the next row in the opposite direction. This alternating scanning sequence enables systematic coverage of the selected slide region and is controlled by a custom Arduino C++ script."

Comment 7: Z-axis focusing and autofocus are not implemented; this limitation should be stated explicitly in the Methods, not only in the Discussion.

Response: Thank you for this clarification. We have now explicitly stated in the Methods section (lines 347–348) that the current ALLocate hardware does not incorporate Z-axis

motorization or autofocus functionality.

“The current implementation does not include Z-axis motorization or autofocus functionality, which will be addressed in future work.”

Comment 8: Reproducibility and methodological detail
At present, the manuscript does not provide enough detail for full reproducibility of the hardware system. Specifically:
• Camera specifications and image acquisition control are not described.
• The computing platform used for image capture and ML inference is not explicitly stated.
• Mechanical coupling, motion resolution, and scan logic of the automated stage are insufficiently detailed.

Providing additional schematics, flow diagrams, or expanded textual descriptions would substantially improve transparency and reproducibility.

Response: Thank you for this important comment regarding reproducibility and methodological detail. We have expanded the Methods section to provide additional information on the hardware configuration, image acquisition, and automated scanning workflow.

Specifically, we have clarified the camera setup and computing platform (see response to **Reviewer 3 Comment 2**), and further detailed the mechanical coupling, stage actuation, firmware control, and tile acquisition strategy (see response to **Reviewer 3 Comment 6**). These additions include descriptions of the raster scanning logic and synchronization between stage motion and image acquisition.

We thank the reviewer again for this comment, which has helped improve the clarity and reproducibility of the manuscript.

Comment 9: Ethics and reporting transparency
Although the data are described as de-identified, the manuscript does not include a clear ethics or IRB statement specifying approval, exemption, or waiver of consent. Given the use of human clinical samples, this information should be explicitly reported.

If a reporting summary or checklist is available, its inclusion would further improve transparency.

> **Response:** We thank the reviewer for highlighting this important omission. This study was conducted in accordance with institutional guidelines and was approved by the Institutional Review Boards of Memorial Sloan Kettering Cancer Center (IRB #16-1591) and the University of California, San Francisco (IRB #2442610).
>
> A reporting summary has been completed and submitted to the journal. We will ensure that the ethics statement and reporting information are explicitly included in the revised manuscript in accordance with editorial requirements.

Overall assessment
This is a strong and timely manuscript with clear potential impact, particularly for low-resource diagnostic settings. The core results are compelling and support publication after minor revision, primarily focused on improving methodological clarity, transparency, and contextualization rather than adding new experimental data.

Comment 10: Code availability

Partially. I inspected the repository contents and README, but I did not execute/install the code end-to-end (the repository does not provide a complete runnable environment, dependencies list, pretrained weights, or example datasets sufficient to reproduce the full pipeline out of the box).

Assessment of the code (reproducibility and community usability)

What is available / positives

The repository is publicly accessible and contains Python code and Jupyter notebooks associated with the ALLocate analysis, including:

a region_cnn/ directory (ROI CNN training assets) and

notebooks for plotting/visualizing performance, plus a central script (ALLocate_software.py).
GitHub

This is a positive step toward transparency, particularly for readers who want to understand or re-create portions of the ML evaluation workflow.

Documentation quality / usability

The README is very brief and currently provides only a high-level statement about where region-CNN training files and plotting/visualization code live. It does not include:

installation instructions (e.g., environment setup/conda/pip),

a requirements.txt/environment specification,

step-by-step commands to run training/inference,

how to obtain/format the datasets,

pretrained weights or model checkpoints,

expected input/output directory structure.
GitHub

There are no releases/packages and no clear “quick start” pathway for new users.
GitHub

Extent to which results are reproducible

Partial reproducibility is feasible for readers already familiar with Python/ML workflows who can infer dependencies and who have access to similar data.

However, full reproducibility of the paper’s end-to-end results is currently limited because the repository does not appear to provide:

the curated annotated datasets used in the paper (or a public substitute with identical preprocessing),

a complete, documented pipeline tying together acquisition → tiling → ROI selection → YOLO inference → slide-level aggregation,

the hardware acquisition/control code (stage/camera integration) and associated configuration details.

Overall judgment

The repository is a useful supplemental resource (especially for understanding some parts of the analysis and plotting), but it is not yet a fully usable community resource for reproducing the complete study without substantial additional documentation and artifacts.

Suggestions to improve code value and reproducibility (for authors)

Add a requirements.txt / environment.yml, plus OS/GPU/CPU notes.

Provide a Quick Start section with exact run commands.

Provide pretrained weights (or instructions to download them) and example config files.

Include a small toy/sample dataset and expected file structure (or scripted download + preprocessing).

Provide an explicit “reproduce paper figures/tables” guide.

If possible, add documentation for the acquisition/control stack (camera control, stage scan logic, synchronization).

Response: Thank you for the careful inspection of the repository and for the constructive recommendations regarding reproducibility and usability. In response to these suggestions, we have substantially expanded the repository to improve clarity and reproducibility. Specifically:

- We have added a requirements.txt file detailing all Python dependencies and tested library versions, as well as the specifications of the computing platforms used in our study and installation instructions.
- A new Quick Start section has been included in the README, providing step-by-step instructions for environment setup, model inference, and reproduction of evaluation outputs. Scripts used to generate certain primary figure panels have been included as well, along with instructions on how to execute them.
- We have documented the expected input/output directory structure and proper configuration settings for running inference.
- Regarding hardware acquisition and stage control, we have included the Arduino firmware and the description of setting up the control architecture to improve transparency of the mechanical scanning subsystem.

Response to the Reviewers

We thank the reviewers for their careful evaluation of our revised manuscript and for their constructive comments. We have addressed all remaining points and have made the corresponding updates to the manuscript, code repository, and Supplementary Information, as detailed below.

Reviewer #1

Remarks to the Author

Reviewer comment:
Thank the authors for the very detailed response. All my concerns have been addressed and I have no further comments.

Response:
We sincerely thank the reviewer for their careful evaluation of our manuscript and for confirming that their concerns have been fully addressed.

Remarks on Code Availability

Reviewer comment:
Package (`tqdm`) is missing from the requirements. Data files are not present for reproducibility (running notebooks).

Response:
We thank the reviewer for identifying these omissions. We have added the `tqdm` package to the requirements file. We have also provided the source data file, which contains the data necessary to run the accompanying notebooks and reproduce the reported analyses.

Reviewer #2

Reviewer comment:
Dear Authors,

Thank you for your comprehensive and carefully detailed reply to the comments. The revisions you have carried out address the main concerns raised during the initial evaluation. In particular, the enlargement of the dataset, the clearer presentation of the methodology, and the

strengthening of the external validation cohort greatly enhance the overall robustness and clarity of the study.

I would also like to emphasize this low-cost microscopic system, based on robust methods with minimal computational requirements, represents a meaningful strength of the study and may offer valuable perspectives for implementation in rapid screening settings, especially in regions or institutions with limited resources.

As you appropriately indicate in the revised manuscript, such an approach still requires expert review to obtain a valuable diagnosis. In this context, I would suggest slightly softening the statement in the abstract. For example: "These results suggest that ALLocate provides an accurate, generalizable, and cost-effective approach bridging the gap between AI innovation and practical deployment for marrow smears screening in underserved regions."

Response:

We sincerely thank the reviewer for their positive assessment of the revised manuscript and for highlighting the potential value of ALLocate in resource-limited settings. We agree that the manuscript should more clearly distinguish the intended screening and triage role of ALLocate from definitive clinical diagnosis.

ALLocate is intended to support rapid bone marrow smear screening in settings where advanced imaging infrastructure and access to specialist expertise may be limited. The system can help identify potentially abnormal cases, quantify blast proportions, and prioritize high-risk cases for further evaluation. However, it is not intended to replace expert assessment for definitive diagnosis.

To address the reviewer's concern and further soften the positioning of the system, we have revised the concluding sentence of the abstract from:

> "These results support the feasibility of low-cost, automated leukemia analysis using conventional microscopes."

to:

> "These results suggest that ALLocate provides an accurate, generalizable, and cost-effective approach for automated bone marrow smear screening, helping bridge the gap between AI innovation and practical deployment in resource-limited settings where access to specialist expertise may be limited."

We have also revised the corresponding statement in the Introduction to avoid presenting ALLocate as a standalone diagnostic system. The revised sentence now reads:

> “Our evaluation demonstrates the robustness and generalizability of ALLocate and supports its potential as a low-cost tool for bone marrow smear screening and triage in resource-limited clinical settings.”

These revisions clarify that ALLocate is intended as an accessible screening and triage tool, particularly in environments with limited specialist resources, while recognizing that definitive diagnosis continues to require expert evaluation.

Reviewer #4

Reviewer comment:
I am invited as a replacement reviewer for previous Reviewer #3, who was not responsive during the revision. I read through previous Reviewer #3’s comments and the responses from the authors. I find that all comments have been properly addressed, except one minor question: the overall specification is much better described, but to really support reproducibility, should the customized 3D-print files also be released and made available as part of the publication?

Response:
We thank the reviewer for this important suggestion. To further support reproducibility and facilitate reconstruction of the ALLocate hardware system, we have provided the customized 3D-print files as Supplementary Files accompanying the revised manuscript. These files contain the designs for the customized 3D-printed components used to assemble the ALLocate system.
